# An epigenetic pathway regulates MHC-II expression and function in B cell lymphoma models

**DOI:** 10.1172/JCI179703

**Published:** 2025-01-16

**Authors:** Te Zhang, Oguzhan Beytullahoglu, Rima Tulaiha, Amanda Luvisotto, Aileen Szczepanski, Natsumi Tsuboyama, Zibo Zhao, Lu Wang

**Affiliations:** 1 Department of Biochemistry and Molecular Genetics and; 2 Simpson Querrey Center for Epigenetics, Feinberg School of Medicine, Northwestern University, Chicago, Illinois, USA.

**Keywords:** Oncology, Epigenetics

## Abstract

Mutations or homozygous deletions of MHC class II (MHC-II) genes are commonly found in B cell lymphomas that develop in immune-privileged sites and have been associated with patient survival. However, the mechanisms regulating MHC-II expression, particularly through genetic and epigenetic factors, are not yet fully understood. In this study, we identified a key signaling pathway involving the histone H2AK119 deubiquitinase BRCA1 associated protein 1 (BAP1), the interferon regulatory factor interferon regulatory factor 1 (IRF1), and the MHC-II transactivator class II transactivator (CIITA), which directly activates MHC-II gene expression. Disruption of the BAP1/IRF1/CIITA axis leads to a functional attenuation of MHC-II expression and MHC-II–dependent immune cell infiltration, leading to accelerated tumor growth in immunocompetent mice. Additionally, we demonstrated that pharmacological inhibition of polycomb repressive complex 1 (PRC1) — which deposits histone H2K119Ub and opposes BAP1 activity — can restore MHC-II gene expression in BAP1-deficient B cell lymphoma cells. These findings suggest that BAP1 may function as a tumor suppressor by regulating the tumor microenvironment and immune response. Our study also establishes the rationale for therapeutic strategies to restore tumor-specific MHC-II expression and enhance immunotherapy outcomes at epigenetic levels in B cell lymphoma treatment.

## Abstract 2

This study has uncovered the molecular mechanisms of how MHC-II molecules were regulated by epigenetic axis and provided novel therapeutic targets for B-cell lymphoma treatment.

## Graphical abstract

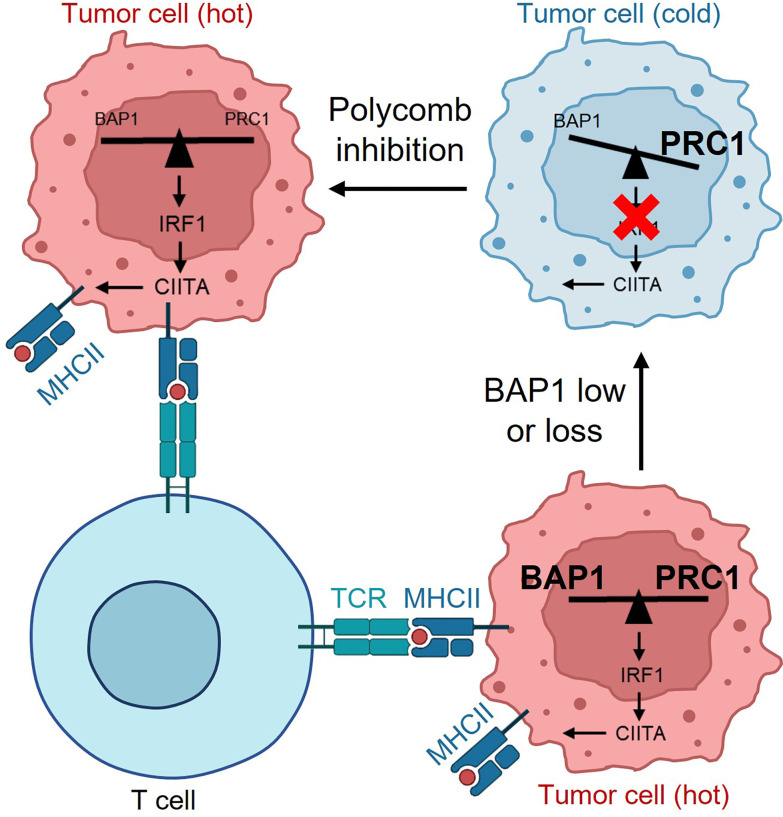

## Introduction

Non-Hodgkin lymphoma (NHL) accounts for 4% of all cancers in the United States, and it was estimated that 20,180 deaths from this disease would occur in the United States in 2023 (1). NHL is a general category of lymphoma that begins in the lymphatic system, part of the body’s germ-fighting immune system (1). Many subtypes fall under the NHL category, and the classification of lymphoma depends on what type of lymphocyte is affected (B cells or T cells), how mature the cells are, when they become cancerous, and other factors (2). B cell lymphoma is a major form of NHL that originates from B cell lymphocytes, which are responsible for producing antibodies in the immune system to combat infections (3, 4). The prevailing subtype of B cell lymphoma is diffuse large B cell lymphoma (DLBCL), a particularly aggressive subtype that continues to be the most common lymphoid malignancy in adults.

The development of B cell lymphoma can be driven by various genetic and molecular alterations; thus, different subtypes of B cell lymphoma have distinct genetic drivers. Several genetic alterations have been identified as playing a role in the development and progression of B cell lymphoma, such as BCL2/BCL6 rearrangement (5) and loss-of-function mutations within tumor suppressor genes such as p53 (6). The current clinical therapy for B cell lymphoma typically involves a combination of chemotherapy, immunotherapy, targeted therapies, or stem cell transplantation (7). However, due to the heterogeneity of B cell lymphoma, high treatment resistance, and relapse ratio (8), the identification of new functional biomarkers to predict the treatment response and outcomes is still an ongoing area of research.

Emerging studies from various research groups have suggested that epigenetic plasticity and reprogramming play a central role in guiding the intricate and dynamic phenotypic shifts observed in B cells during both immune response and lymphomagenesis (9). Research has demonstrated that disruptions in the B cell lymphoma epigenome and the biological consequences of changes in epigenetic factors contribute to lymphoma progression. Examples of these contributions include gain-of-function mutations in EZH2 (10, 11), loss-of-function mutations in MLL4/KMT2D (12, 13), frequent genetic abnormalities affecting histone acetyltransferases CBP and p300 (14), and abnormal DNA methylation patterns (15). Collectively, epigenetic lesions hold promise as biomarkers to enhance the accuracy of diagnosis and prognosis in B cell lymphoma (9). Alongside conventional chemotherapy and immunotherapy approaches, epigenetic therapies are gaining momentum in light of such findings. Further research is necessary to fully comprehend the impact of the newly identified epigenetic alterations on the genesis of B cell lymphoma, as they have the potential to provide novel insights and advance the development of improved biomarkers and therapies (16).

The BRCA1 associated protein 1, also known as BAP1, is a major epigenetic factor that forms a multiprotein complex with ASXL1-3, FOXK1/2, MBD5/6, HCF1, and OGT (17) and functions as a general transcriptional activator by catalyzing the removal of mono-ubiquitin from histone H2AK119 at both enhancer (18) and promoter regions (19). Depletion of BAP1 leads to a genome-wide increase of mono-ubiquitination levels of histone H2AK119, which is deposited by the polycomb repressive complex 1 (PRC1) (20, 21). In both mouse embryonic stem cells and human cancer cell lines, the transcriptional defect induced by BAP1 depletion or catalytic-inactive mutations could be fully rescued by PRC1 depletion (21, 22). Therefore, the precise actions between the BAP1 complex and its opposing complex, PRC1, maintain the equilibrium of chromatin state and gene expression. Notably, the recent development of BAP1 inhibitors and PRC1 inhibitors by our laboratory (22, 23) and other groups (24, 25) allows us to understand further how the E3 ligase activity of PRC1 and deubiquitinase activity of BAP1 on histone H2AK119 governs the balance of chromatin states and proper transcriptional programming in the context of normal and tumorigenic states. It has been demonstrated that the dysregulation of BAP1 and its associated factors are involved in the tumorigenesis of many different cancer types, including uveal melanoma (26), breast cancer (27, 28), prostate cancer (29, 30), leukemia (19, 21), and lung cancer (31, 32). However, to date, the role of BAP1 in regulating B cell lymphoma development remains to be elucidated.

The tumor microenvironment (TME) is composed of both proinflammatory and immunosuppressive cells and influences the overall immune state of the tumor (33). Patients with a greater population of cytotoxic lymphocytes (CTL) tend to have more favorable outcomes relative to those with a higher proportion of immunosuppressive cells, who succumb to earlier disease recurrence (34). Multiple factors influence the activity of CTLs in the TME. For instance, the MHC encodes a series of proteins that are responsible for eliciting a cellular immune response (35). The classical MHC class II (MHC-II) proteins are expressed constitutively on antigen-presenting cells including monocytes, macrophages, dendritic cells, and B cells, and they are responsible for presenting peptide antigens derived from exogenous proteins to T cells (36). MHC-II is critical for antigen presentation to CD4^+^ T lymphocytes, whose role in antitumor immunity is becoming increasingly noted, and decreased MHC-II molecule expression has been demonstrated to be associated with poor survival in patients with B cell lymphoma (37). In the current study, we will elucidate how BAP1 is involved in transcriptional regulation of MHC-II expression and how BAP1 impacts B cell lymphoma tumor growth via regulating TME and immune cell infiltration.

## Results

### BAP1 regulates MHC-II cluster genes in a catalytically dependent manner.

**Figure 1:**
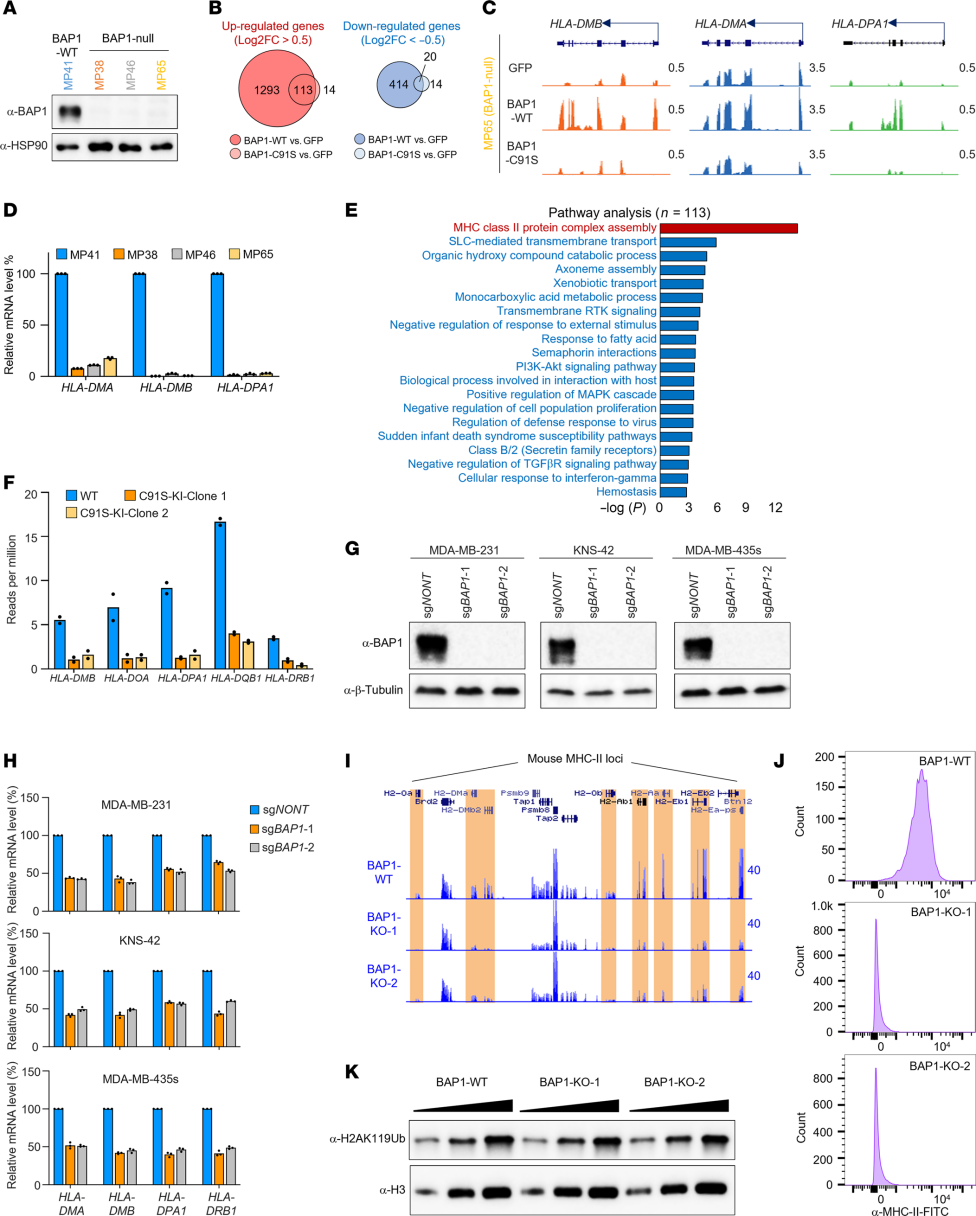
BAP1 regulates MHC-II cluster genes in a catalytically dependent manner. (**A**) The protein levels of BAP1 in 4 uveal melanoma cell lines MP41, MP38, MP46, and MP65 were determined by Western blot. Representative blot from 2 biological repeats. (**B**) The Venn diagram shows the overlap between WT and catalytically dead BAP1 rescued genes in BAP1-null MP65 cell line. (**C**) The track example shows the expression levels of MHC-II cluster genes in MP65 BAP1 null cells restored by GFP, BAP1-WT, or BAP1-C91S. (**D**) The mRNA levels of MHC-II cluster genes were determined by real-time PCR in MP41, MP38, MP46, and MP65 cells. *n* = 3 technical replicates. Data are represented as mean ± SD. (**E**) Pathway analysis was performed with the genes that are selectively upregulated by WT BAP1 but not catalytically dead BAP1 in BAP1-KO HEK293T cells. (**F**) The mRNA levels of MHC-II cluster genes were determined by RNA-Seq in BAP1-WT and catalytic-dead KI HEK293T cells. BAP1 was depleted by CRISPR with 2 distinct sgRNAs in MDA-MB-231, KNS42, and MDA-MB-435s cell lines. (**G**) The protein levels of BAP1 were determined by Western blot. Representative blot from 2 biological repeats. (**H**) The mRNA levels of MHC-II cluster genes *HLA-DMA*, *HLA-DMB*, *HLA-DPA1*, and *HLA-DRB1* were determined by real-time PCR. *n* = 3 technical replicates. Data are represented as mean ± SD. (**I**) The track example shows the mRNA levels of MHC-II gene loci in BAP1-WT and BAP1-KO A20 cells. (**J**) The protein levels of cell-surface MHC-II molecule in BAP1-WT and -KO A20 cells were determined by FACS and analyzed by FlowJo software. (**K**) The protein levels of histone H2AK119Ub were determined by Western blot in both the WT A20 and 2 independent BAP1-KO clones. Representative blot from 2 biological repeats.

It has been demonstrated that BAP1 regulates context-dependent transcriptional programs in different cancer types and may play opposing roles in different tumorigenesis (38). For instance, recurrent loss-of-function mutations within BAP1 have been detected in uveal melanoma (26), indicating a potential tumor-suppressive function of BAP1 in this specific cancer type. To dissect how BAP1 impacts the tumor-suppressive transcriptional program in cancer cells, we examined 4 different human uveal melanoma cell lines including BAP1 WT (MP41) and null cells (MP38, MP46, and MP65) (Figure 1A). To investigate how the deubiquitinase activity of BAP1 is involved in the transcriptional regulation in uveal melanoma cells, we reexpressed either WT or catalytically dead (C91S) BAP1 in the BAP1-null MP65 cell line (Supplemental Figure 1A; supplemental material available online with this article; https://doi.org/10.1172/JCI179703DS1). As shown in the RNA-Seq data, reexpression of WT BAP1 leads to an upregulation of 1,406 genes and downregulation of 434 genes, while the catalytically dead (C91S) BAP1 has a very mild impact on gene expression (Figure 1B), indicating the gene-expression regulation by BAP1 is mostly catalytic dependent. Notably, several MHC-II cluster genes were identified as the top upregulated genes by WT but not the catalytically dead BAP1 (Figure 1C). Intriguingly, these genes were all remarkably decreased in BAP1-null uveal melanoma cells as compared with BAP1 WT cells (Figure 1D). Next, to further understand whether BAP1 regulates the MHC-II cluster genes in a cell type–dependent manner, we reexpressed WT or catalytically dead BAP1 in BAP1-KO HEK293T cells (Supplemental Figure 1B) followed by RNA-Seq analysis (Supplemental Figure 1C). As a result, we found the MHC-II cluster genes were the top enriched gene cluster in WT but not C91S mutant BAP1-reexpressed HEK293T cells (Figure 1E and Supplemental Figure 1D). To further strengthen this conclusion, we introduced the catalytically dead mutation (C91S) by CRISPR knockin (KI) in HEK293T cells (Supplemental Figure 1, E and F) and compared the gene expression patterns between WT and C91S-KI cells by RNA-Seq. As shown in Figure 1F, we found a substantial reduction of MHC-II gene expression in the 2 independent C91S-KI clones. Furthermore, to determine whether BAP1 regulates the expression of MHC-II cluster genes in different cancer types, we depleted BAP1 in breast cancer cell line MDA-MB-231, brain tumor cell line KNS-42, and melanoma cell line MDA-MB-435s by CRISPR (Figure 1G). Then we assessed the mRNA levels of the highly expressed MHC-II cluster genes in these cell lines by utilizing real-time PCR assay. As shown in Figure 1H, a reduction of MHC-II cluster genes was observed in all BAP1-depleted cell lines from 3 different cancer types. Besides the human cancer cell lines, we also knocked out BAP1 in a mouse B cell lymphoma cell line A20, which constitutively overexpresses MHC-II cluster genes (Supplemental Figure 1G). We assessed both the mRNA and protein levels of MHC-II cluster genes in BAP1-WT and depleted A20 cells by RNA-Seq and FACS analysis. Consistently, BAP1 deficiency strongly reduced the constitutively overexpressed MHC-II genes in A20 murine B cell lymphoma cells (Figure 1, I and J, and Supplemental Figure 1H) without upregulating the bulk H2AK119Ub levels (Figure 1K). Finally, we analyzed the RNA-Seq data from an independent study in which WT BAP1 was reexpressed in uveal melanoma UM22 (BAP1 null) cell lines (39). As shown in Supplemental Figure 1I, most of the MHC-II cluster genes but not MHC-I cluster genes were dramatically upregulated by BAP1 reexpression. Collectively, these results indicated that BAP1 may directly regulate MHC-II cluster gene expression, and this regulatory effect is independent of cell types.

### BAP1 occupies MHC-II gene loci and governs chromatin states.

**Figure 2:**
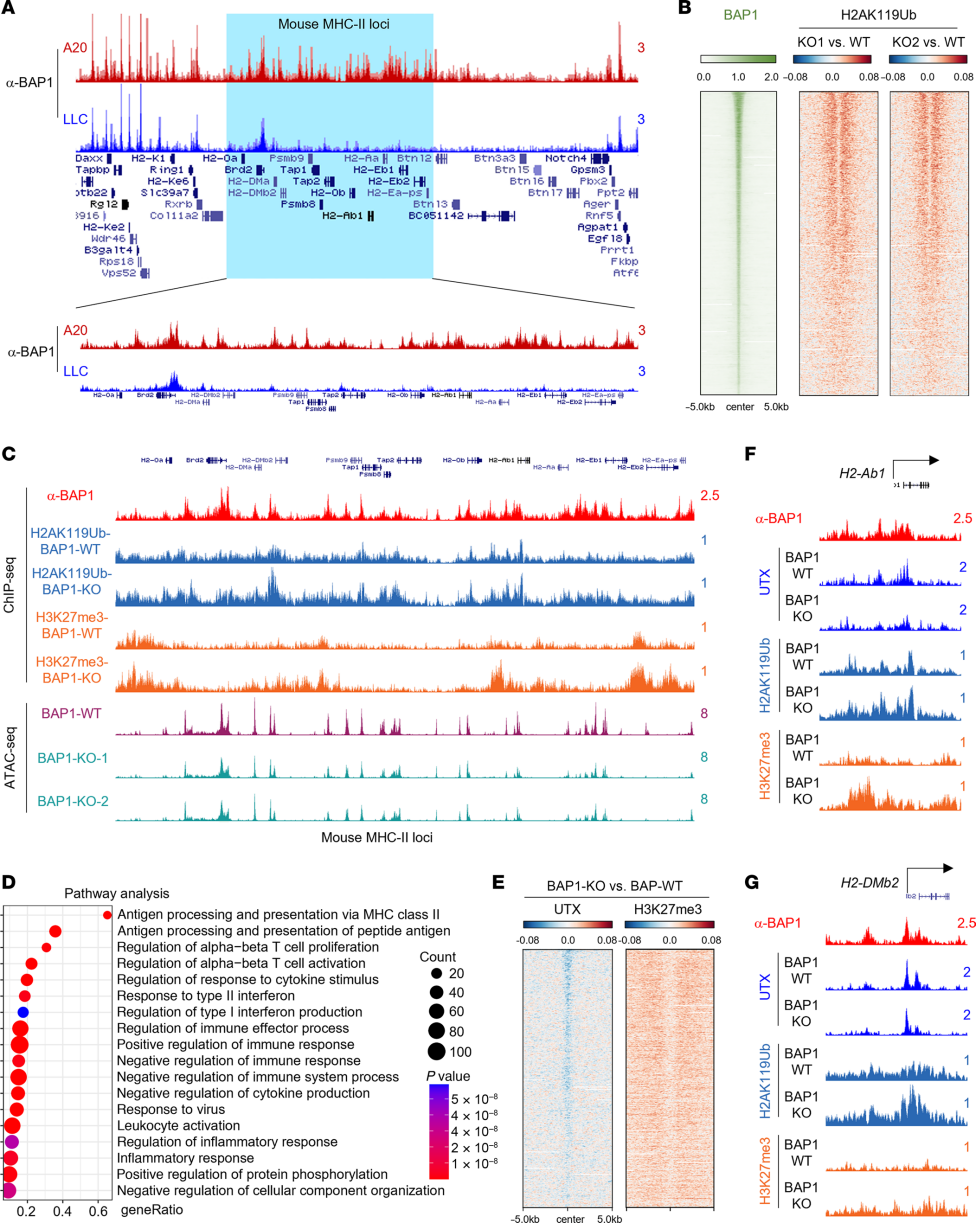
BAP1 occupies MHC-II gene loci and governs chromatin states. (**A**) The track example shows BAP1 occupancy at the MHC-II gene loci in MHC-II–positive cell line A20 cells and MHC-II–negative cell line LLC cells. (**B**) The heatmap shows the log_2_ fold change of H2AK119Ub levels in BAP1-WT and 2 independent KO clones. All of the ChIP-Seq signals were centered on BAP1 peaks in BAP1-WT cells. (**C**) The track example shows the H2AK119Ub and H3K27me3 levels between BAP1-WT and -KO A20 cells (upper panel). The bottom panel shows the ATAC-Seq signal from both BAP1-WT and 2 independent KO clones at MHC-II gene loci. (**D**) Pathway analysis with the genes that are downregulated in BAP1-KO A20 cells. (**E**). The heatmap shows the log_2_ fold change of UTX occupancy and H3K27me3 levels in BAP1-WT and -KO A20 cells centered on BAP1 peaks. (**F** and **G**) The track examples show the reduction of UTX occupancy and the increase of H3K27me3 levels at MHC-II cluster genes in BAP1-WT and -KO A20 cells.

Based on the cell line gene expression profile retrieved from the Cancer Cell Line Encyclopedia (CCLE) database (40), lymphocytes express the highest levels of MHC-II cluster genes (Supplemental Figure 2, A and B). Therefore, we decided to utilize the A20 cell line, a murine B lymphoma cell line derived from a BALB/c mouse with spontaneous reticulum cell neoplasms (41), for the following studies. To explore how BAP1 regulates MHC-II gene expression at the chromatin levels, we conducted BAP1 ChIP-Seq with a validated BAP1 ChIP-Seq antibody (Supplemental Figure 2C) in the A20 cell line with the mouse LLC cell line as negative control. The LLC cell line is an MHC-II–negative cell line, but it expresses levels of BAP1 protein equal to those of the A20 cell line (Supplemental Figure 2D). As a result, we detected a high occupancy level of BAP1 at the MHC-II cluster regions in the A20 cell line, compared with LLC cells (Figure 2A). Since BAP1 depletion does not affect bulk H2AK119Ub levels in A20 cells (Figure 1K), we conducted H2AK119Ub ChIP-Seq to determine whether loss of BAP1 affects loci-specific H2AK119 levels. As a result, BAP1 depletion leads to a dramatic increase in H2AK119Ub levels at BAP1-occupied loci, at both the genome-wide level (Figure 2B) and the MHC-II gene loci-specific level (Figure 2C). This result reveals that the histone H2AK119Ub deubiquitination activity of BAP1 may also be involved in regulating the expression of MHC-II cluster genes in B cell lymphoma cells as in other cell types (Figure 1). Indeed, consistent with the results we obtained from mouse A20 cells, depletion of BAP1 also leads to an increase in H2K119Ub levels in HEK293T cells at the human MHC-II gene loci (Supplemental Figure 2E). In support of our hypothesis that the chromatin-bound BAP1 at MHC-II gene loci is critical for the transcriptional regulation of gene expression, ATAC-Seq analysis revealed that depletion of BAP1 strongly reduced the chromatin accessibility at the MHC-II cluster region in the A20 cell line (Figure 2C). Notably, the pathway analysis with all the downregulated genes in the BAP1-depleted A20 cell line shows that the MHC-II cluster gene–associated pathway is top ranked (Figure 2D).

We and others have previously shown that the BAP1 complex was responsible for the recruitment of MLL3/UTX-COMPASS to catalyze the removal of H3K27me3 at genome-wide levels (18). To further clarify the underlying impact of BAP1 on regulating UTX recruitment and histone H3K27me3 levels, we conducted ChIP-Seq analysis with UTX and H3K27me3-specific antibodies in BAP1-WT/KO A20 cells. As shown in Figure 2, E–G, we found that depletion of BAP1 leads to a dramatic reduction of UTX occupancy and increased H3K27me3 levels at BAP1-occupied loci (Figure 2E), such as MHC-II cluster genes (Figure 2, F and G, and Supplemental Figure 2F). Previous studies published by Levine’s group (42) and others have shown that loss of BAP1 function can result in EZH2-dependent hematopoietic transformation in vivo. Based on our independent analysis, BAP1 deficiency in myeloid progenitors led to a dramatic increase of H3K27me3 levels at the MHC-II loci (Supplemental Figure 2G), and a substantial reduction in MHC-II gene expression (Supplemental Figure 2, H and I), which is consistent with our conclusions.

### Loss of BAP1 attenuates IRF1-dependent regulation of the CIITA/MHC-II axis.

**Figure 3:**
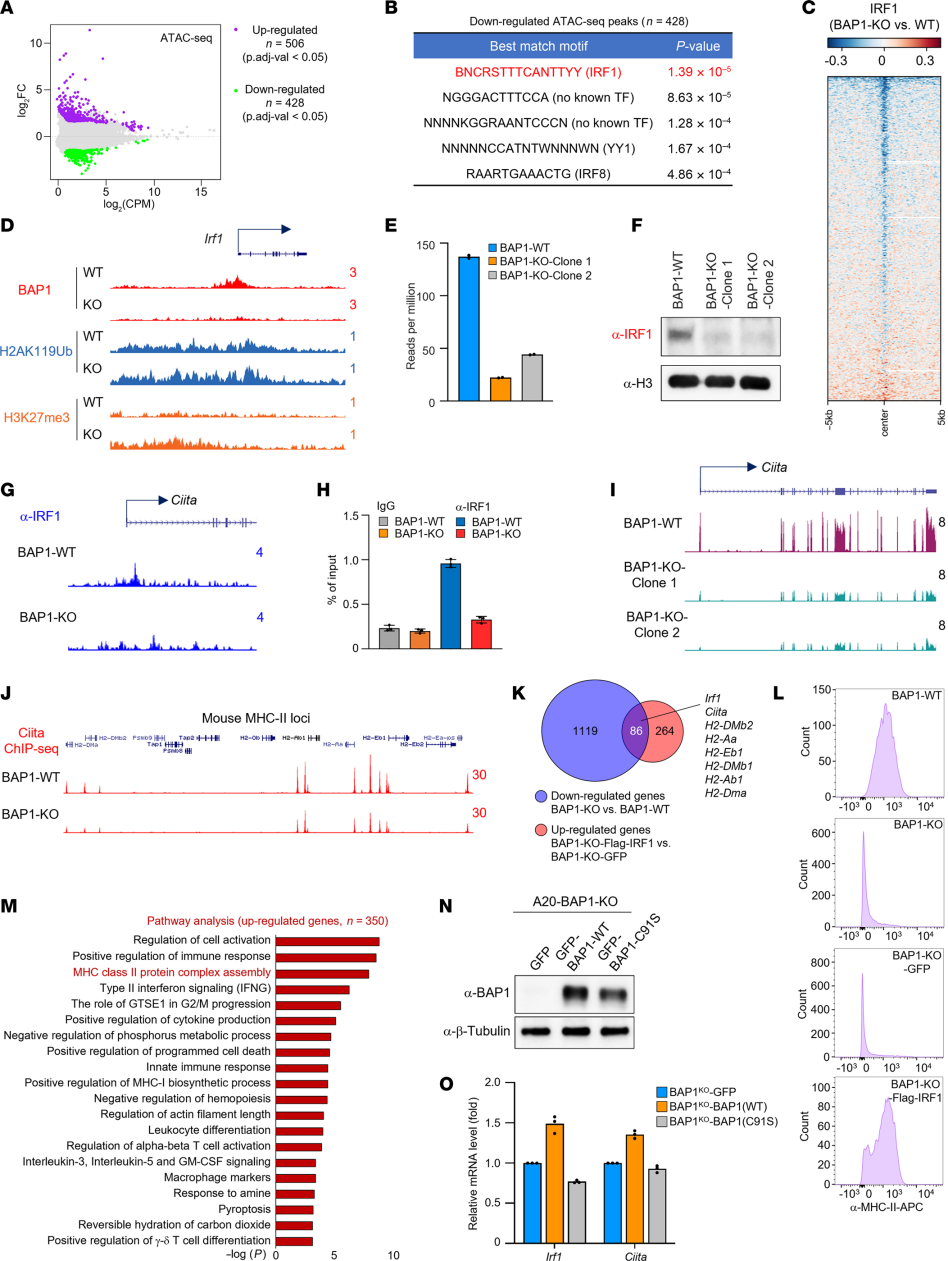
Loss of BAP1 attenuates IRF1-dependent regulation of CIITA/MHC-II axis. (**A**) The MA plot (Bland-Altman plot) shows the up- or downregulated ATAC-Seq signal comparing BAP1-WT/KO cells. (**B**) The motif analysis with the downregulated ATAC-Seq peaks in BAP1-KO cells. (**C**) The heatmap shows the log_2_ fold change of IRF1 chromatin binding in BAP1-WT and -KO cells. (**D**) The track example shows the chromatin occupancy of BAP1 and the protein levels of H2AK119Ub and H3K27me3 at the *Irf1* gene locus. (**E**) The mRNA levels of *Irf1* in A20 BAP1-WT and -KO cells were determined by RNA-Seq. (**F**) The protein levels of IRF1 in A20 BAP1-WT and -KO cells were determined by Western blot. Representative blot from 2 biological repeats. (**G**) The track example shows the chromatin occupancy of IRF1 at the *Ciita* promoter in both BAP1-WT and -KO cells. (**H**) The occupancy of IRF1 at the *Ciita* promoter region was quantified by ChIP-QPCR in BAP1-WT and -KO cells. *n* = 3 technical replicates. Data are represented as mean ± SD. (**I**) The track example shows the *Ciita* levels in BAP1-WT and -KO cells. (**J**) The track example shows the occupancy of CIITA at MHC-II cluster gene loci in BAP1-WT and -KO cells. (**K**) The Venn diagram shows the genes that are rescued by IRF1 in BAP1-KO cells. (**L**) The protein levels of MHC-II molecules in BAP1-WT, BAP1-KO, BAP1-KO-GFP, and BAP1-KO-Flag-IRF1 cells were determined by FACS analysis. (**M**) The pathway analysis with the total upregulated genes by IRF1 in A20 BAP1-KO cells. The BAP1-KO A20 cells were transduced with either GFP, WT BAP1, or catalytically dead BAP1. (**N**) The protein levels of BAP1 were determined by Western blot. Representative blot from 2 biological repeats. (**O**) The mRNA levels of *Irf1* and *Ciita* were determined by real-time PCR. *n* = 3 technical replicates. Data are represented as mean ± SD.

To further uncover the molecular mechanisms underlying BAP1-dependent transcriptional regulation and chromatin states, we compared the ATAC-Seq results between BAP1-WT and -KO A20 cells. Overall, we detected 506 and 428 peaks that were up- and downregulated in BAP1-depleted cells, respectively (Figure 3A). Next, we performed motif analysis by GREAT software (43) with the 428 downregulated ATAC-Seq peaks and identified the interferon regulatory factor 1 (IRF1) motif as the top hit (Figure 3B), while no significant enriched motifs were found in the 506 upregulated peaks in the absence of BAP1. To further validate the ATAC-Seq results, we conducted ChIP-Seq analysis to determine IRF1 occupancy in both BAP1-WT and -KO cells. As a result, we detected a dramatic decrease in IRF1 occupancy across the genome when BAP1 was depleted (Figure 3C). Interestingly, based on our ChIP-Seq analysis, BAP1 directly binds to the promoter region of the mouse *Irf1* gene, and BAP1 deficiency leads to a dramatic increase of both H2AK119Ub and H3K27me3 levels at the *Irf1* gene loci (Figure 3D). Indeed, BAP1 depletion results in a subsequent reduction in *Irf1* mRNA levels (Figure 3E) and protein levels (Figure 3F). As one of the major transcription factors, IRF1 directly binds to the promoter region and transcribe class II transactivator (CIITA) (44), which subsequently initiates MHC-II cluster gene expression (45). As expected, the reduced IRF1 levels at the *Ciita* promoter in BAP1-KO cells (Figure 3, G and H) leads to a strong repression of *Ciita* gene expression (Figure 3I) and subsequently a reduction of CIITA protein that occupies the MHC-II cluster gene loci (Figure 3J). Moreover, to validate whether the BAP1/IRF1/CIITA axis is a major regulator of MHC-II genes’ expression in B cell lymphoma, we restored IRF1 in BAP1-KO A20 cell (Supplemental Figure 3A) followed by RNA-Seq analysis and FACS analysis. As shown in Figure 3, K and L, we found both *Ciita* and the key MHC-II cluster genes, such as *H2-DMb2*, *H2-Aa*, *H2-Eb1*, *H2-DMb1*, *H2-Ab1*, and *H2-DMa*, could be fully rescued by reexpression of IRF1 in BAP1-KO cells. Consistent with the RNA-Seq results, the pathway analysis shows that the rescued genes by IRF1 in BAP1-KO cells are enriched in MHC-II gene pathways (Figure 3M and Supplemental Figure 3B). To study whether the BAP1 activity is involved in the transcriptional regulation of the *Irf1* gene, we reexpressed either WT or catalytically dead BAP1 in BAP1-KO A20 cells (Figure 3N) and assessed the mRNA levels of *Irf1* and *Ciita* by real-time PCR. Indeed, only the WT BAP1 was capable of rescuing *Irf1* and *Ciita* gene expression in BAP1-KO cells (Figure 3O). These results demonstrated that the BAP1/IRF1/CIITA axis is critical for maintaining MHC-II cluster gene expression in B cell lymphoma cells.

### Pharmacological inhibition of PRC1 activity restores the transcriptional defect induced by BAP1 loss.

**Figure 4:**
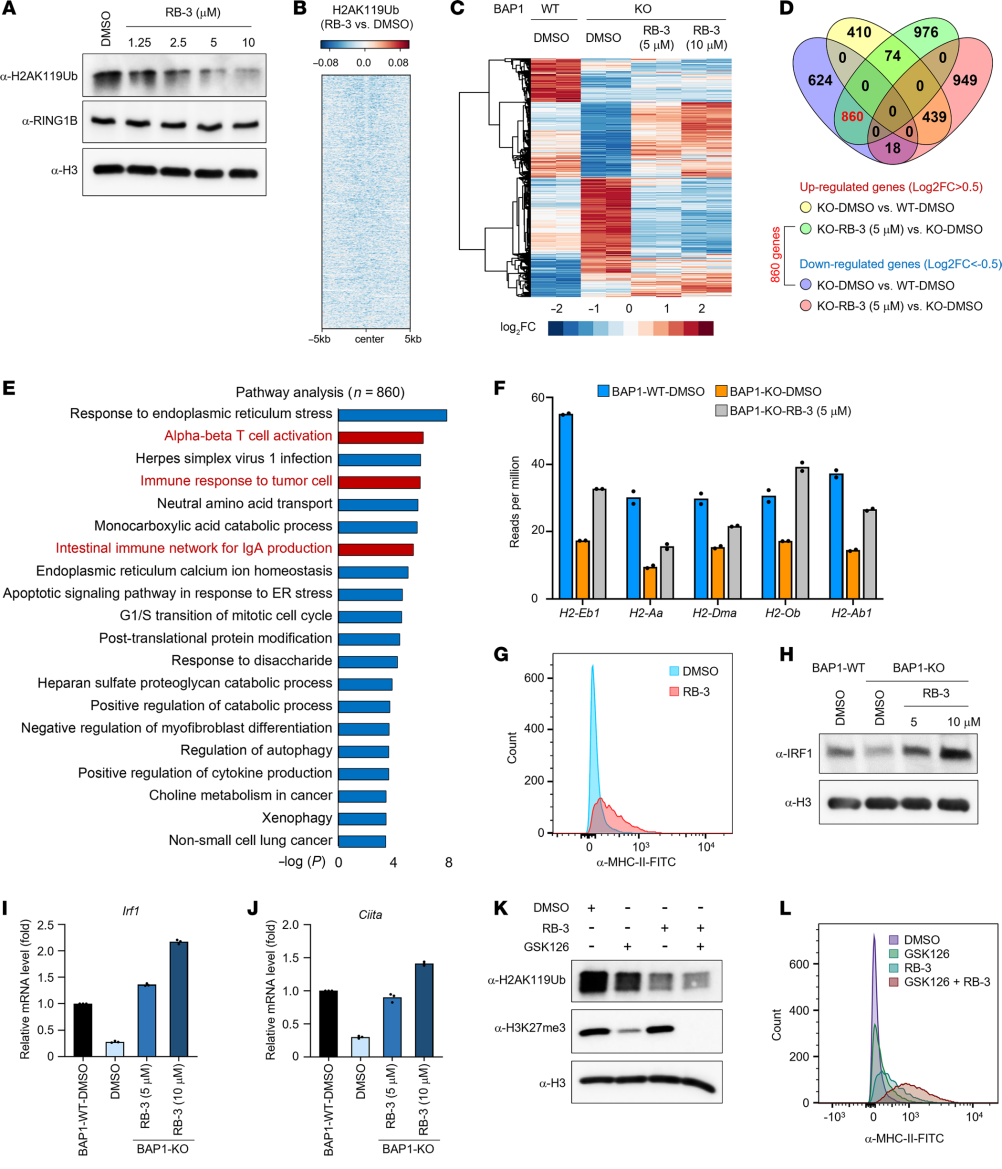
Pharmacological inhibition of PRC1 activity restores the transcription defect induced by BAP1 loss. (**A**) The A20 BAP1-KO cells were treated with various concentrations of PRC1 inhibitor RB-3 for 6 days. The protein levels of H2AK119Ub and RING1B were determined by Western blot. Representative blot from 2 biological repeats. (**B**) The log_2_ fold change heatmap shows the H2AK119Ub levels in A20 BAP1-KO cells treated with either DMSO or RB-3. (**C**) The A20 BAP1-WT or -KO cells were treated with either DMSO or RB-3 for 6 days. The gene-expression profiles for each condition were determined by RNA-Seq. (**D**) The Venn diagram shows the overlap between BAP1 targeted genes and RB-3 rescued genes. (**E**) The pathway analysis with the 860 BAP1 targeted genes that were restored by RB-3 treatment. (**F**) The A20 BAP1-WT cells were treated with DMSO for 6 days, and the BAP1-KO cells were treated with either DMSO or RB-3 (5 μM) for 6 days. The mRNA levels of MHC-II cluster genes in each treatment were determined by RNA-Seq. (**G**) The BAP1-KO A20 cells were treated with either DMSO or RB-3 (10 μM) for 6 days. The protein levels of MHC-II were determined by FACS analysis. (**H**) The A20 BAP1-WT cells were treated with DMSO for 6 days, and the BAP1-KO cells were treated with either DMSO or various concentrations of RB-3 for 6 days. The IRF1 protein levels were determined by Western blot. Representative blot from 2 biological repeats. (**I** and **J**) The mRNA levels of *Irf1* (**I**) and *Ciita* mRNA (**J**) were determined by real-time PCR. *n* = 3 technical replicates. Data are represented as mean ± SD. (**K** and **L**) The BAP1-KO cells were treated with RB-3 (10 μM) or/and GSK126 (5 μM) for 6 days. The protein levels of H2AK119Ub and H3K27me3 were determined by Western blot. (**K**) Representative blot from 2 biological repeats. The protein levels of cell surface MHC-II molecules were determined by FACS analysis (**L**).

It has been well documented that the BAP1 complex functions as a major deubiquitinase of histone H2AK119, which is catalyzed by the PRC1 (20, 21). Initially, both our group and others have shown that the transcriptional defect induced by loss of BAP1 could be fully rescued by PRC1 depletion (21, 22, 46). Therefore, we sought to determine whether the pharmacological inhibition of PRC1 activity could rescue the MHC-II cluster genes in BAP1-KO cells. The small molecule inhibitor RB-3 has been designed and synthesized as a chemical probe that is able to disrupt the association of RING1B-BMI1 with chromatin and thereby inhibit H2A ubiquitination in human acute myeloid leukemia (AML) cells (24). Interestingly, we found the protein levels of H2AK119Ub were decreased in a dose-dependent manner in BAP1-KO A20 cells treated with various concentrations of the PRC1 inhibitor RB-3 (Figure 4A). This result was further validated by ChIP-Seq analysis, which showed a reduction of H2AK119Ub levels at genome-wide levels upon RB-3 treatment (Figure 4B). Subsequently, in order to verify whether RB-3 could rescue the transcriptional defect in the BAP1-KO A20 cell line, we treated the BAP-WT and -KO A20 cell lines with either DMSO or various concentrations of RB-3, followed by RNA-Seq analysis. As shown in Figure 4, C and D, a large number of genes (*n* = 860) downregulated in BAP1-KO cells were found to be rescued by RB-3 treatment. Notably, those genes were enriched in multiple immune response and T cell function pathways (Figure 4E). The vast majority of the detectable MHC-II cluster genes were partially rescued by RB-3 treatment (Figure 4, F and G). As we expected, as a major downstream transcriptional target of BAP1, both the mRNA and the protein levels of IRF1 in BAP1-KO cells could be fully rescued by RB-3 treatment to the same level or exceeding that of WT cells (Figure 4, H and I). Consistently, the mRNA levels of *Ciita* were also upregulated upon the RB-3 compound treatment (Figure 4J). Interestingly, we also noticed that depletion of IRF1 in BAP1-KO cells was not able to completely abolish the induction of *Ciita* expression upon PRC1 inhibition (Supplemental Figure 4, A and B), indicating the small molecule inhibitor RB-3 could activate MHC-II gene expression via targeting both BAP1-dependent and -independent pathways.

It has been demonstrated that H2AK119Ub deposition is essential to maintain PRC2 function and PcG-target gene repression in multiple cell types (47). Loss of H2AK119Ub will lead to a rapid displacement of PRC2 activity and a loss of H3K27me3 deposition (46). In addition, recent studies have shown that treatment of the EZH2 inhibitor contributes to the elevation in MHC-II expression in mice and activation of infiltrating T cells (48, 49). Therefore, we endeavored to further investigate the impact of the PRC1 inhibitor on H3K27me3 levels and MHC-II gene expression in B cell lymphoma cells. As shown in Supplemental Figure 4C, we found treatment of the PRC1 inhibitor RB-3 could also lead to a depression of H3K27me3 at genome-wide levels. Meanwhile, treatment of the EZH2 inhibitor, GSK126, functions similarly to the PRC1 inhibitor on gene expression and MHC-II cluster gene expression in BAP1-KO cells (Supplemental Figure 4, D and E). Specifically, we detected 1,005 genes (e.g. *Irf1*, *Ciita*, and MHC-II genes) that were repressed by BAP1 depletion and could be rescued by the treatment of either RB-3 (PRC1 inhibitor) or GSK126 (PRC2 inhibitor) (Supplemental Figure 4, F and G). Finally, to determine a potential combination effect of both PRC1 and PRC2 inhibitors on reactivation of MHC-II gene expression in BAP1-deficient B cell lymphoma cells, we treated BAP1-KO A20 cells with RB-3 or/and GSK126 for 6 days, followed by Western blot and FACS analysis. As a result, we found that the combination treatment of both inhibitors could completely abolish H3K27me3 levels in cells (Figure 4K) and dramatically increase the MHC-II protein levels (Figure 4L). Our study has revealed a potential combination therapy with polycomb inhibitors for the treatment of B cell lymphoma.

### Loss of BAP1 reduces immune cell infiltration and accelerates B cell lymphoma growth in vivo.

**Figure 5:**
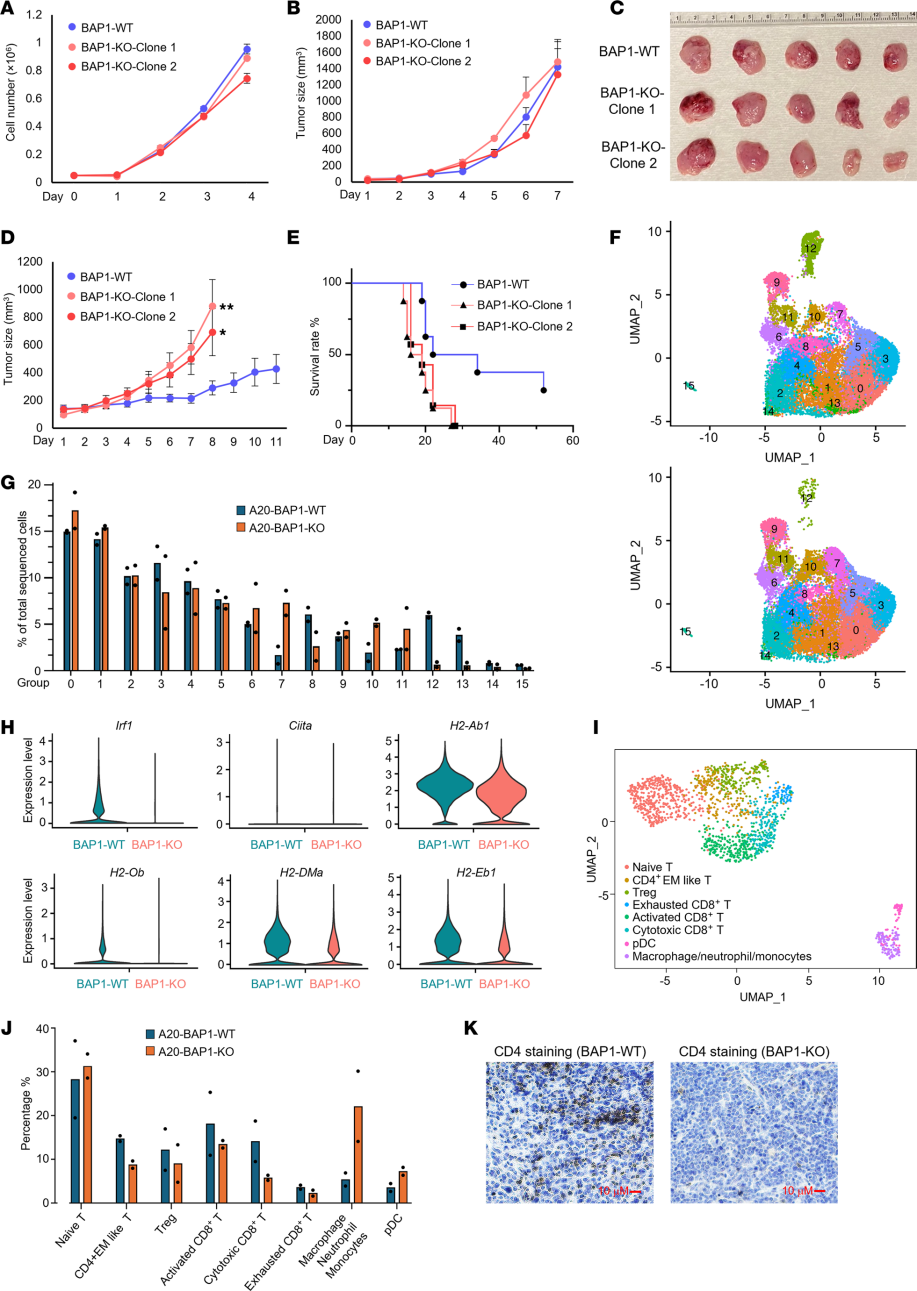
Loss of BAP1 reduces immune cell infiltration and promotes B cell lymphoma growth in vivo. (**A**) The cell growth rate of A20 BAP1-WT and -KO cells was determined by cell counting assay. (**B**) 2 × 10^6^ of BAP1-WT and BAP1-KO clones were inoculated into the right flank of 6-week-old nude mice. The tumor size was measured every day for 1 week after inoculation. (**C**) Images of representative tumor tissue samples from each mouse were taken at the end of the experiment. (**D**) 5 × 10^6^ of BAP1-WT and BAP1-KO clones were inoculated into the right flank of 6-week-old BALB/c mice. The tumor size was measured every day for 2 weeks after the inoculation. Data are represented as mean ± SEM. A 2-tailed unpaired Student’s *t* test was used for statistical analysis. **P < 0.01; *P < 0.05. (**E**) When each tumor reached 1 cm^3^, the mouse was euthanized, and the survival probability was shown. (**F**) Tumor tissue from both BAP1-WT and BAP1-KO was harvested and subjected to scRNA-Seq. The UMAP analysis identifies different clusters of cell populations within the tumor tissue based on the gene-expression profiles. (**G**) The bar plot shows the percentage of each cell cluster in the tumor tissues. (**H**) The violin plot shows the expression levels of *Irf1*, *Ciita*, and MHC-II cluster genes in BAP1-WT and -KO tumor cell population. (**I**) Immune cells from the first clustering were isolated (T cells and macrophages) and reclustered with UMAP algorithm. (**J**) The percentages of each type of immune cells were calculated in BAP1-WT and -KO A20 tumors and are shown in the bar plot. (**K**) The IHC staining was performed with CD4-specific antibody in BAP1-WT and -KO tumor tissues. Scale bars: 10 μm.

BAP1 has been known as a common essential factor, the depletion of which leads to cell death and apoptosis in many cell types, including both normal and cancer cells (21, 22). However, in B cell lymphoma cells, a complete knockout of BAP1 has no significant effect on cell proliferation in vitro (Figure 5A) or in immunocompromised mice (Figure 5, B and C). Consistently, based on our RNA-Seq analysis shown in Figure 2D, depletion of BAP1 has minimal impact on cell cycle or proliferation genes. Instead, it leads to a dysregulation of genes involved in immune response and microenvironment. Therefore, to study how BAP1 impacts TME and tumor growth, we inoculated BAP1-WT and -KO A20 cells, which were originally derived from BALB/c mice, into normal BALB/c mice. Surprisingly, depletion of BAP1 significantly accelerated tumor growth in BALB/c mice (Figure 5D) and shortened the animal survival rate (Figure 5E). To further determine how loss of BAP1 accelerates B cell lymphoma cells growth in vivo via regulating immune cell infiltration and TME, we conducted single-cell RNA-Seq (scRNA-seq) with the tumor samples from both BAP1-WT and -KO tissues (Supplemental Figure 5A). As shown in Figure 5F, we have analyzed the gene-expression profiles from 2 BAP1-WT tumor tissues (WT-1:11,393 cells; WT-2: 11,384 cells), and 2 BAP1-KO tumor tissues (KO-1: 10,166 cells; KO-2: 10,881 cells) and in total identified 16 cell clusters (no. 0 to approximately no. 15) based on the gene-expression profiles. Notably, we identified cluster no. 12, which specifically expresses T cell markers, as the most markedly decreased population in BAP-KO tumor tissues (Figure 5G and Supplemental Figure 5, B and C). Interestingly, the second most remarkable reduced cell population, cluster no. 15, specifically carried macrophage markers, indicating that loss of BAP1 may also lead to an infiltration defect of innate immune cells (Figure 5G and Supplemental Figure 5F). To validate whether loss of BAP1 leads to similar gene-expression repression as in vitro experiments, we analyzed the expression levels of *Irf1*, *Ciita*, and MHC-II cluster genes between BAP1-WT and -KO tumors. The scRNA-Seq analysis suggested a gene expression pattern similar to that seen in in vitro RNA-Seq results (Figure 5H and Supplemental Figure 5, D–G). Next, we sought to determine whether the increased tumor growth ability is due to the loss of MHC-II molecules in BAP1-deficient A20 cells. We depleted CIITA by CRISPR in BAP1-WT cells (Supplemental Figure 5, H and I) and confirmed a dramatic decrease in MHC-II expression by FACS analysis (Supplemental Figure 5J). Intriguingly, we have detected a significant reduction of tumor growth in the absence of CIITA/MHC-II expression in an animal model (Supplemental Figure 5K). This result highlights a potential role of CIITA/MHC-II involved in BAP1-dependent B cell lymphoma growth in animals. Finally, we conducted immune cell profiling with our scRNA-Seq results (Figure 5I) and identified that the CD4^+^ effector memory–like (EM-like) T cell is the only immune cell population that is substantially reduced in BAP1-KO tumor tissues (Figure 5J and Supplemental Figure 5L). Given that it is well established that CD4^+^ T cells generally recognize MHC-II molecules, we further confirmed the attenuated CD4^+^ T cell infiltration by IHC staining (Figure 5K). Together, these results demonstrated that tumor-infiltrating immune cells were attenuated by loss of BAP1, promoting B cell lymphoma proliferation in vivo.

### Epigenetic balance established by BAP1/PRC1 in transcriptional regulation and TME.

**Figure 6:**
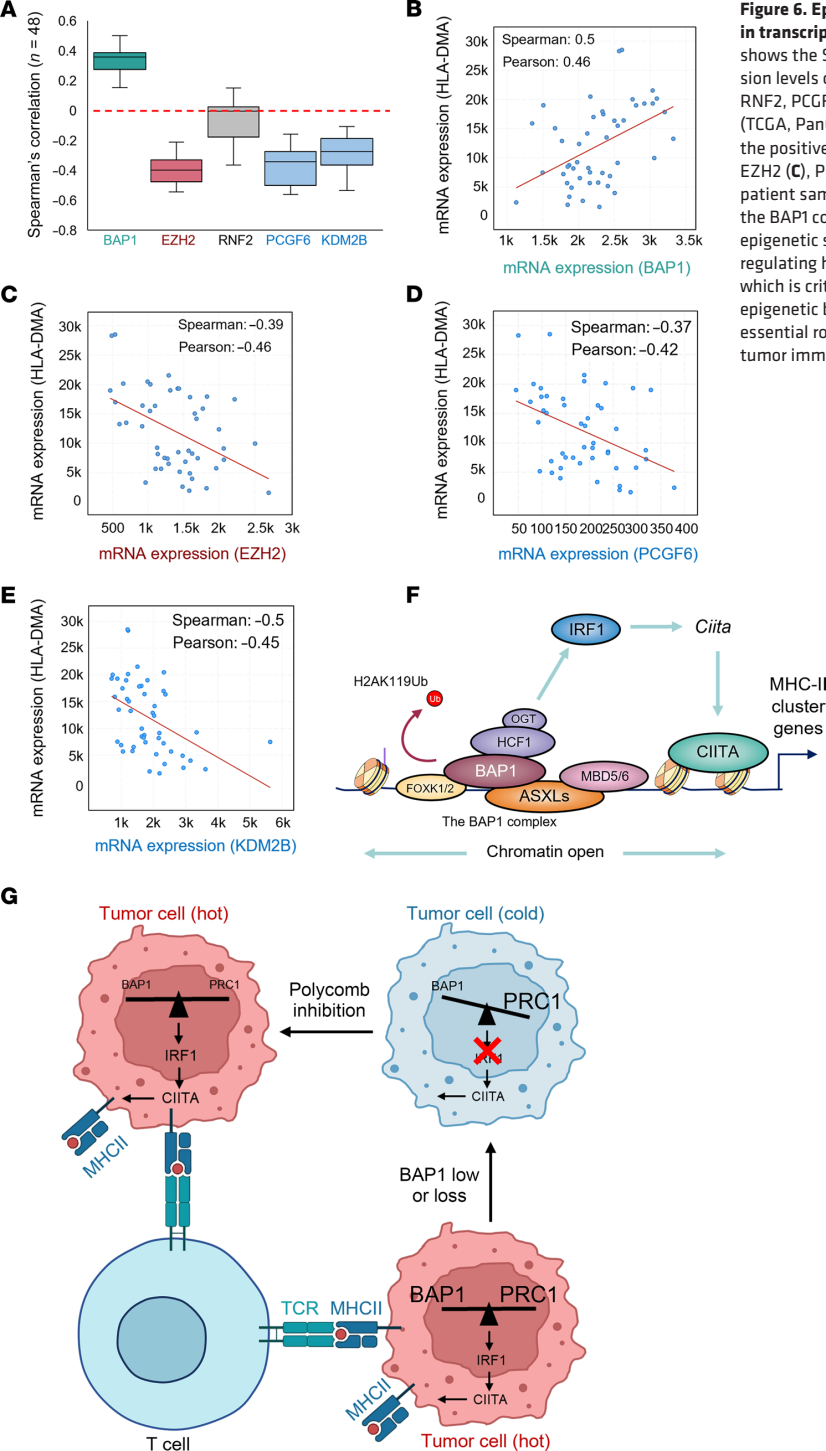
Epigenetic balance established by BAP1/PRC1 in transcriptional regulation and TME. (**A**) The box plot shows the Spearman’s correlation between the expression levels of human MHC-II genes and BAP1, EZH2, RNF2, PCGF6, or KDM2B in B cell lymphoma patients (TCGA, PanCancer Atlas). (**B**–**E**) The scatter plot shows the positive correlation between HLA-DMA and (**B**), EZH2 (**C**), PCGF6 (**D**), or KDM2B (**E**) in B cell lymphoma patient samples. (**F**) The schematic model shows how the BAP1 complex and the PRC1 establish a dynamic epigenetic state at MHC-II cluster gene loci by precisely regulating histone H2AK119Ub level and IRF1/CIITA axis which is critical for MHC-II gene expression. (**G**) The epigenetic balance between BAP1 and PRC1 plays an essential role in “hot” and “cold” tumor switching and tumor immune response.

Finally, to determine the impact of BAP1 and polycomb balance on MHC-II gene expression in DLBCL patient samples, we employed The Cancer Genome Atlas (TCGA) dataset, containing the gene-expression data of 48 DLBCL patient samples, for additional validation. As a result, we observed a positive correlation between BAP1 and MHC-II cluster gene-expression levels, while EZH2 (catalytic subunit within PRC2) showed a negative correlation with MHC-II gene expression (Figure 6A). Interestingly, there was only a mild negative correlation between RNF2 (catalytic subunit within PRC1) and MHC-II cluster gene expression, suggesting only a subset of PRC1 subcomplexes are involved in the transcriptional repression of MHC-II genes (50). Indeed, we detected a stronger negative correlation between MHC-II gene expression and 2 key subunits of noncanonical PRC1, PCGF6, and KDM2B (Figure 6, A–E). These results indicate that a critical epigenetic balance established between BAP1 and polycomb is critical for maintaining proper expression levels of MHC-II cluster genes in B cell lymphoma cells.

It has been well documented that polycomb functions as a major antagonist of BAP1 in mammalian cells. The inhibition of PRC1 by its small molecular inhibitor leads to a reactivation of the IRF1/CIITA axis and a remarkable reduction of H2AK119Ub and H3K27me3 levels at the MHC-II gene loci in BAP1-null cells. This results in the restoration of functional MHC-II molecules in tumor cells (Figure 6F). Loss-of-function mutations within BAP1 and its subunits were frequently observed in many types of cancers, which may contribute to the development of resistance to immunotherapy. Hence, the precise and dynamic balance between BAP1 and PRC1 is imperative for maintaining MHC-II gene expression and orchestrating the switch between the “hot” or “cold” status of tumor cells (Figure 6G). Consequently, the reestablishment of equilibrium in BAP1/polycomb proteins through the use of small molecule inhibitors or degraders targeting polycomb proteins may hold the key to reversing immunotherapy resistance.

## Discussion

Emerging studies have revealed both the oncogenic and tumor-suppressive function of BAP1 in mammalian cells (17). As an oncogenic epigenetic factor, BAP1 has been shown to facilitate tumor metastasis via stabilizing KLF5 in triple-negative breast cancer (51). In addition, BAP1 is essential in prostate cancer (29), small cell lung cancer (31), and mouse melanoma (52) cell viability in vitro and in vivo. On the other hand, BAP1 also possesses tumor-suppressive functions by participating in DNA repair processes, ensuring the damaged DNA is correctly repaired before cell division (53). When BAP1 functions properly, it helps maintain genomic stability and prevents the accumulation of mutations that could drive cancer development. Loss-of-function mutations within subunits of the BAP1 complex were observed in several types of cancer, such as leukemia (19) and uveal melanoma (26), supporting a potential tumor-suppressive function of BAP1. In normal B cell development, BAP1 transcriptionally controls the cell-cycle progression program and is critical for B lymphocyte viability (54). Interestingly, in our current studies, we found that BAP1 controls different transcriptional programs in B cell lymphoma other than in normal B cells. Notably, the depletion of BAP1 does not affect cell-cycle or proliferation genes and has a mild effect on B cell lymphoma cell viability in vitro. However, the loss of BAP1 leads to a substantial acceleration of B cell lymphoma cell growth in immunocompetent mice, indicating that BAP1 may function as a tumor suppressor via regulating TME and tumor immune response in this context.

Decrease or loss of MHC-II expression in B cell lymphoma has been associated with genetic alterations, such as CIITA deficiency, and is associated with poor survival in human B cell lymphoma. The downregulation or the loss of MHC-II molecules has been observed in B cell lymphoma, and mutations or homozygous deletions of MHC-II genes are frequently encountered in lymphomas that develop in immune-privileged sites. The restoration or induction of MHC-II expression holds remarkable importance in activating CD4^+^ helper T cells, thereby enabling cytotoxic CD8^+^ T cells to exert their effectiveness. The loss of MHC-II expression in DLBCL has been associated with genetic alterations, including the overexpression or gain-of-function mutations in polycomb proteins such as EZH2 (49), the enzymatic subunit within PRC2. As the principal polycomb repressive complex, PRC1 also plays a pivotal role in maintaining the repressed chromatin state and PRC2 function by monoubiquitinating histone H2A. In our current studies, we demonstrated that PRC1 is responsible for the transcriptional repression of MHC-II genes upon BAP1 depletion, as inhibition of PRC1 could largely rescue the gene-expression defect in BAP1-depleted B cell lymphoma cells. Loss of BAP1 leads to a dramatic reduction of MHC-II gene expression and reduced immune cell infiltration in animal models, resulting in a substantial acceleration of tumor growth. Notably, we discovered that BAP1 tends to function as a tumor suppressor in hot tumors with high MHC-II expression (Supplemental Figure 2A), such as lymphoma, blood cancer (19, 42), and skin cancers (55). In contrast, it may show oncogenic function in cold tumors with low MHC-II molecule expression such as small cell lung cancer (31), prostate cancer (29), and breast cancer (51). Therefore, cancer patients with low or absent antitumor immunity respond to immune checkpoint inhibitor treatment may benefit from the pharmacological inhibition of BAP1 activity.

Our current studies have suggested that the precise regulation of MHC-II cluster genes is established by the histone H2AK119Ub deubiquitinase BAP1 and PRC1 at 2 distinct layers. First, BAP1 positively regulates the expression of *Irf1*, which serves as a direct transcription factor of *Ciita*. The latter encodes CIITA, an indispensable factor for the expression of MHC-II cluster genes. Additionally, BAP1 occupies the MHC-II cluster gene loci, ensuring the maintenance of chromatin accessibility in a catalytic-dependent manner. It has been well documented that multiple signaling/factors are involved in the transcriptional or posttranslational control of IRF1 (56, 57) and CIITA (58–61) in mammalian cells. Therefore, it is difficult to determine to what degree the changes in MHC-II expression are a direct consequence of BAP1 regulation of IRF1 as compared with direct BAP1 loading and regulation of MHC-II genes. Nevertheless, our study demonstrated that the loss of BAP1 disrupts the IRF1/CIITA axis, along with the proper chromatin states at MHC-II cluster gene loci, culminating in a repression of MHC-II gene expression and function.

Emerging research underscores the crucial involvement of the enzymatic functions of PRC1 core components, RING1B, in the development and progression of various types of tumors. To date, both RING1B inhibitors (24) and PRC1 degraders (62) have been developed and tested in various human cancer cell line models. However, there is limited understanding of the impact of PRC1 inhibitors on MHC-II gene expression and the TME. In our current studies, we demonstrated that inhibition of PRC1 activity by its specific small molecule inhibitor RB-3 could at least partially rescue the transcriptional defect induced by BAP1 depletion in B cell lymphoma cells. Consistent with the results from the initial work that generated this small molecule inhibitor (24) we found RB-3 only functions at high concentrations in vitro (~10 μM), which limits its efficacy in the in vivo experiments. Future studies will be focused on improving the specificity and efficacy of this small molecule inhibitor in vivo.

Epigenetic silencing has been identified as a key regulatory mechanism affecting MHC-II expression in B cell lymphoma. Loss-of-function mutations in epigenetic activators such as MLL4 COMPASS (13), the SWI/SNF complex (63), and CREBBP (64), along with hyperactivations of epigenetic repressors such as EZH2 (65) and HDAC3 (66) are frequently observed in B cell lymphoma, often resulting in the silencing of MHC-II cluster genes. Notably, the inhibition of transcriptional repressors such as EZH2 (67, 68) and HDAC3 (66, 69) has been investigated as a strategy to restore MHC-II expression in B cell lymphoma models. Additional optimizations may be required to improve the potency and efficacy of these small molecule inhibitors.

In summary, our studies have uncovered a potential role of histone H2AK119 deubiquitinase BAP1 and the epigenetic balance between PRC1 and BAP1 in regulating MHC-II gene expression and TME in B cell lymphoma cells. In addition, our work has elucidated the context-dependent oncogenic/tumor-suppressive function of BAP1 and highlighted the potential of therapeutically attenuating PRC1 activity in BAP1-deficient cold tumors.

## Methods

### Sex as a biological variable.

Given the disease etiology, sex was not considered as a biological variable.

### Mouse experiments.

Mice were housed 5 per cage and maintained under specific pathogen–free conditions. Twelve hours of light were provided each day. Food and water were provided freely. Five- to 6-week-old Balb/c mice and nude mice purchased from Jackson lab were used for xenograft experiments. The tumor growth was measured every day using a calibrated caliper 2 weeks after inoculation. A 2-tailed unpaired Student’s *t* test was used for statistical analysis.

### Antibodies and reagents.

H3K27me3 (catalog 9733), Histone H3 (catalog 4499S), H2AK119Ub (catalog 8240), IRF1 (catalog 8478S), RING1B (catalog 5694S), UTX (catalog 33510S), CD4 (catalog 25229S), MHC-II-FITC (catalog 42594S), MHC-II-APC (catalog 64776), and BAP1 (catalog 13271) antibodies were purchased from Cell Signaling Technology. Tubulin antibody (E7) was purchased from Developmental Studies Hybridoma Bank. HSP90 (sc-7947), GFP (sc-9996), and GAPDH (sc-32233) antibodies were purchased from Santa Cruz Biotechnology Inc. The BAP1- and CIITA-specific ChIP-grade polyclonal antibodies were generated in house.

### Cell lines.

The MDA-MB-231, MDA-MB-435S, and HEK293T cells were purchased from ATCC and maintained with DMEM (Fisher Scientific, 15013CV) containing 10% FBS (MilliporeSigma). The MP41, MP38, MP46, MP65, LLC, and A20 cell lines were obtained from ATCC and were maintained with ATCC-formulated RPMI 1640 medium containing 10% FBS (MilliporeSigma). The KNS-42 cell line was a gift from Rintaro Hashizume (Department of Pediatrics, the University of Alabama at Birmingham, Birmingham, Alabama, USA).

### IP.

The IP experiments were performed as previously described (31). Briefly, the cells were lysed in the lysis buffer (50 mM Tris, pH 8.0, 150 mM NaCl, 0.5% Triton X-100, 10% glycerol, protease inhibitors, and benzonase). After centrifugation at maximum speed (20,000*g*) at 4°C for 15 minutes, the supernatant was collected and incubated with the antibody and protein A/G beads (Santa Cruz) at 4°C overnight with rotation. Then the protein A/G beads were washed with ice-cold lysis buffer 4 times and boiled in 5× SDS sample loading buffer.

### Oligos used for constructs and real-time PCR.

Designed sgRNAs were cloned into either lentiCRISPR, version 2 (Addgene, 52961) vector. The lentiviral-mediated CRISPR/Cas9 knockout was described previously (18). Oligo sequences used in this manuscript were as follows: sg*NONT* (GCTGAAAAAGGAAGGAGTTGA), sg*BAP1*-1 (human) (CACGGACGTATCATCCACCA), sg*BAP1*-2 (human) (GAACCGTCAGACAGTACTAG), sg*BAP1*-1 (mouse) (ACCTGTCTGAGTGCACTCAG), sg*BAP1*-2 (mouse) (TCAGCTATGTGCCTATCACA), and sgIRF1 (mouse) (CGGAGCTGGGCCATTCACAC). The clone ID for mouse *Ciita* was TRCN0000086452, and the nontargeting (sh*NONT*) shRNA construct (SHC002) was purchased from MilliporeSigma. Primer sequences for real-time PCR used in this manuscript were as follows: *H2Aa*_F: 5′-TCAAATTCCACCCCAGCTAC-3′, *H2Aa*_R: 5′-CTATTTCTGAGCCATGTGATGTTG-3′; *H2Ab1*_F: 5′-GATCTTCCTCGGGCTTGG-3′, *H2Ab1*_R: 5′-ATTCGGAGCAGAGACATTCAG-3′; *H2Eb1*_F: 5′-CTGTGATCCTGTTGCTGACA-3′, *H2Eb1*_R: 5′-AGTATCTTTCCAGAAGCCGC-3′; *H2DMa*_F: 5′-TCCTAGAGAATGCCCTGTGT-3′, *H2DMa*_R: 5′-TGTGCTGTTCCAAGATCTCC-3′; *H2DMb1*_F: 5′-AGCCCTAACCCCTTCCTAC-3′, *H2DMb1*_R: 5′-GCAGACACAGAGACCTTCAC-3′; *H2DMb2*_F: 5′-GTGAAGGTCTCTGTGTCTGC-3′, *H2DMb2*_R: 5′-TTCTGCTTTCTAGTGCCGTC-3′; *H2Oa*_F: 5′-CCGTAATGAGCTTCCTGAGTC-3′, *H2Oa*_R: 5′-TGTTCCCCGTCAAATTCGTG-3′; *H2Ob*_F: 5′-CCTTTGTCCCCTCCAGAATG-3′, *H2Ob*_R: 5′-TGAAGTAACAGTCCGCCTTTG-3′; m*Ciita*_F 5′-GATCCTTCCAGCCTTCTCTTC-3′, m*Ciita*_R 5′-TTGTCTCCGATCTTGTTCTCAC-3′; m*Irf1*_F: 5′-GAAGGGAAGATAGCCGAAGAC-3′, m*Irf1*_R: 5′-TCTGGTTCCTCTTTGCAGC-3′; m*Gapdh*_F: 5′-AACAGCAACTCCCACTCTTC-3′, m*Gapdh*_R: 5′-CCTGTTGCTGTAGCCGTATT-3′; *HLA-DMA*_F: 5′-CCAGCAAATAGGGCCAAAAC-3′, *HLA-DMA*_R: 5′-GGGTGGGAAGAGATTACTGAC-3′; *HLA-DMB*_F: 5′-AACTCCCGGCATCTTTACAG-3′, *HLA-DMB*_R: 5′-TGAAATCCTTTGGAGTCCCAG-3′; *HLA-DOA*_F: 5′-CAGAGTGTAATGGCCCTCAG-3′, *HLA-DOA*_R: 5′-CGCCGTAAGACTGGTAGAAG-3′; *HLA-DPA1*_F: 5′-ACAGAATGTTCCATATCAGAGCTG-3′, *HLA-DPA1*_R: 5′-CCTGTTGGTCTATGCGTCTG-3′; *HLA-DQB1*_F: 5′-CCTTCGTCTCAGTTATGTCTTGG-3′, *HLA-DQB1*_R: 5′-TGCCCTTAAACTGGAACACG-3′; *HLA-DRB1*_F: 5′-CAGAATGGAGACTGGACCTTC-3′, *HLA-DRB1*_R: 5′-TGTGCAGATTCAGACCGTG-3′; h*GAPDH*_F: 5′-GTCTCCTCTGACTTCAACAGCG-3′, and h*GAPDH*_R: 5′-ACCACCCTGTTGCTGTAGCCAA-3′. Primer sequences for ChIP-QPCR used in this manuscript were as follows: *H2-DMA*-_F: 5′-CAGGGGACTCAACAGAGCAG-3′, *H2-DMA*-_R: 5′-ATGGTTGCTTGCCATTCGTG-3′; *H2-DMB2*-_F: 5′-CAAAGGAGGGGCTGAGTGAC-3′, *H2-DMB2*-_R: 5′-CAAGGGCCTCAGGTTCTCTC-3′; *H2-Ab1*-_F: 5′-GAAGACTCCTGCATGGGGAA-3′, *H2-Ab1*-_R: 5′-TGCAAGCCATGTTCCCTGAA-3′; *H2-Eb1*-_F: 5′-CCCATCTGCACAGCTCAAGA-3′, *H2-Eb1*-_R: 5′- TGCAGAGCAGTGGACAAACA -3′; and *Ciita*-_F: 5′-ACCAAACACCTGCCTTGGAA-3′, *Ciita*-_R: 5′-TAGGGAGTATCTGTGGCGCT-3′.

### RNA-Seq and analysis.

RNA-Seq was conducted as previously described (31). All the steps for library construction were used according to the manufacturer’s recommendations. Samples were pooled and sequenced on a HiSeq with a read length configuration of 150 PE. Gene counts were computed by HTSeq and used as an input for edgeR 3.0.8. Genes with Benjamini-Hochburg adjusted *P* values of less than 0.01 were considered to be differentially expressed.

### ChIP-Seq assay and analysis.

ChIP-Seq was performed as described previously (31). The cell pellets were collected and washed twice with ice-cold PBS and then fixed with 1% paraformaldehyde for 10 minutes at room temperature. Then the paraformaldehyde solution was quenched with 2.5 M glycine, and the cell pellets were washed twice with PBS afterwards. The cell pellets were resuspended with lysis buffer (50 mM HEPES, pH = 7.5, 140 mM NaCl, 1 mM EDTA, 10% glycerol, 0.5% NP-40, 0.25% Triton X-100, 1× protease inhibitors) and incubated on a nutator for 10 minutes in the cold room. Afterwards, the cell pellets were centrifuged at 500*g* for 5 minutes and the supernatant was discarded. Then, the cell pellets were washed with wash buffer (10 mM Tris-HCl, pH = 8.0, 200 mM NaCl, 1 mM EDTA, 0.5 mM EGTA, 1× protease inhibitors) and resuspended with the sonication buffer (10 mM Tris-HCl, pH = 8.0, 1 mM EDTA, 0.1% SDS, 1× protease inhibitors). Sonication was performed with 1 ml Covaris tubes which were set to 5%–20% duty factor, 175 peak intensity power, and 200 cycles per burst for 120–600 seconds. The 10× dilution buffer (10% Triton X-100, 1 M NaCl, 1% Na-Deoxycholate, 5% *N*-lauroylsarcosine, 5 mM EGTA) was further added to the lysate, and samples were centrifuged at maximum speed for 15 minutes at 4°C. The antibody was added (~ 5 μg commercial antibody or 40 μl of homemade anti-sera) to each sample. After incubation at 4°C overnight, 100 μl protein A/G agarose beads were added for 4 hours. Then the beads were washed 4 times with RIPA buffer (50 mM HEPES, pH = 7.5, 500 mM LiCl, 1 mM EDTA, 1.0% NP-40, 0.7% Na-Deoxycholate), followed by once with ice-cold TE buffer (with 50 mM NaCl). The DNA for each IP sample was eluted with elution buffer (50 mM Tris-HCl, pH = 8.0, 10 mM EDTA, 1.0% SDS) and reverse cross-linked in a 65°C oven for 13 hours, followed by protease K digestion at 55°C for 2 hours. The genomic DNA fragments were then further purified with a QIAGEN DNA Purification Kit (catalog 28104). For ChIP-Seq data analysis, all the peaks were called with the MACS, version 2.1.0, software using default parameters and corresponding input samples. Metaplots and heatmaps were generated by utilizing ngsplot database to display ChIP-Seq signals. Peak annotation, motif analysis, and super enhancer analysis were performed with HOMER (http://homer.ucsd.edu/homer/motif/) and ChIPseeker (https://bioconductor.org/packages/release/bioc/html/ChIPseeker.html). Pathway analysis was performed with Metascape (https://metascape.org/gp/index.html#/main/step1).

### ATAC-Seq and analysis.

ATAC-Seq was performed as described previously (70). Briefly, the frozen cells were thawed and washed once with PBS and then resuspended in-cold ATAC lysis buffer. The cell number was further calculated by Cellometer Auto 2000 (Nexcelom Bioscience). For each sample, 50K to 100K nuclei were centrifuged (prechilled) at 500*g* for 10 minutes. The supernatant was then removed, and the nuclei were resuspended in 50 μl of tagmentated DNA. The reactions were then incubated at 37°C for 30 minutes in a thermomixer shaking at 1,000 rpm, and then cleaned up by the MiniElute Reaction Clean Up Kit (QIAGEN). Tagmentated DNA was amplified with barcode primers. Library quality and quantity were assessed with Qubit 2.0 DNA HS Assay (Thermo Fisher), Tapestation High Sensitivity D1000 Assay (Agilent Technologies), and QuantStudio 5 System (Applied Biosystems). Equimolar pooling of libraries was performed based on quality control (QC) values and sequenced on an Illumina HiSeq (Illumina) with a read length configuration of 150 PE for 50M PE reads (25M in each direction) per sample. ATAC-Seq reads were shifted + 4 bp and − 5 bp for positive and negative strands, respectively, using the alignmentSieve function from deepTools package. ATAC-Seq peaks were called with Macs, version 2.1.0.

### scRNA-Seq.

Cells were washed and resuspended in PBS plus 0.04% BSA. The quantity and quality of the cells were accessed by acridine orange and propidium iodide dye on a Cellometer Auto 2000 (Nexcelom). Dead cells were then removed by the Dead Cell Removal Kit (Miltenyi Biotec). Around 20K cells with viability higher than 70% were loaded on a 10× Chromium Controller based on 5′ Chromium Single Cell Reagent Kit manual (10x Genomics). After cell partitioning and gel bead-in-emulsion (GEM) generation, reverse transcription was performed, and cDNA was pooled and cleaned up by beads. cDNA was further amplified for 14 cycles and cleaned up by SPRI beads (Beckman). The quality of cDNA was assessed by High Sensitivity D5000 Tapestation (Agilent Technologies Inc.) and quantified by Qubit, version 2.0, DNA HS assay (Thermo Fisher). 5′ Gene expression library preparation was carried out according to the 5′ Chromium Single Cell Reagent Kit manual (10x Genomics). Equimolar pooling of libraries was performed based on QC values and sequenced on an Illumina NovaSeq (Illumina) with a read length configuration of 150 PE for 500 M PE reads per sample (250M in each direction).

### Statistics.

For statistical analyses, GraphPad Prism 7, Microsoft Excel, and R were used. All data involving a statistical analysis being reported met the criteria to use the appropriate statistical tests; for the normal distribution of data, the empirical rule was used to infer the distribution. For growth curves and time-course, RNA-Seq *t* tests were calculated between the AUC values. Statistical tests used are reported in the figure legends.

### Study approval.

The research activities with vertebrate animals were conducted in the IACUC and Association for Assessment and Accreditation of Laboratory Animal Care–approved (AAALAC-approved) Center for Comparative Medicine (CCM) facilities. This study was approved by Northwestern University Institutional Animal Care and Use Committee (animal protocol No. IS00013610). All animal studies were conducted in compliance with the ethical guidelines.

### Data availability.

The raw and processed next-generation sequencing data generated in this study have been deposited to the NCBI’s Gene Expression Omnibus database (GEO GSE276031). Values for all data points in graphs are reported in the Supporting Data Values file.

## Author contributions

LW designed the study. LW, TZ, OB, and RT performed the biochemical experiments. TZ performed all the animal experiments. OB and AS performed FACS analysis. ZZ performed the bioinformatics analysis. RT and NT performed technical and material support duties. LW, RT, and AL revised the manuscript.

## Supplementary Material

Supplemental data

Unedited blot and gel images

Supporting data values

## Version 1 01/16/2025

Electronic publication
