# Supplementary material for: An epigenetic pathway regulates MHC-II expression and function in B cell lymphoma models
Source: J Clin Invest. 2025 Jan 16;135(2):e179703. doi: 10.1172/JCI179703 (PMC11735100; doi:10.1172/JCI179703)
Supplement: Supplemental data [file jci-135-179703-s146.pdf]

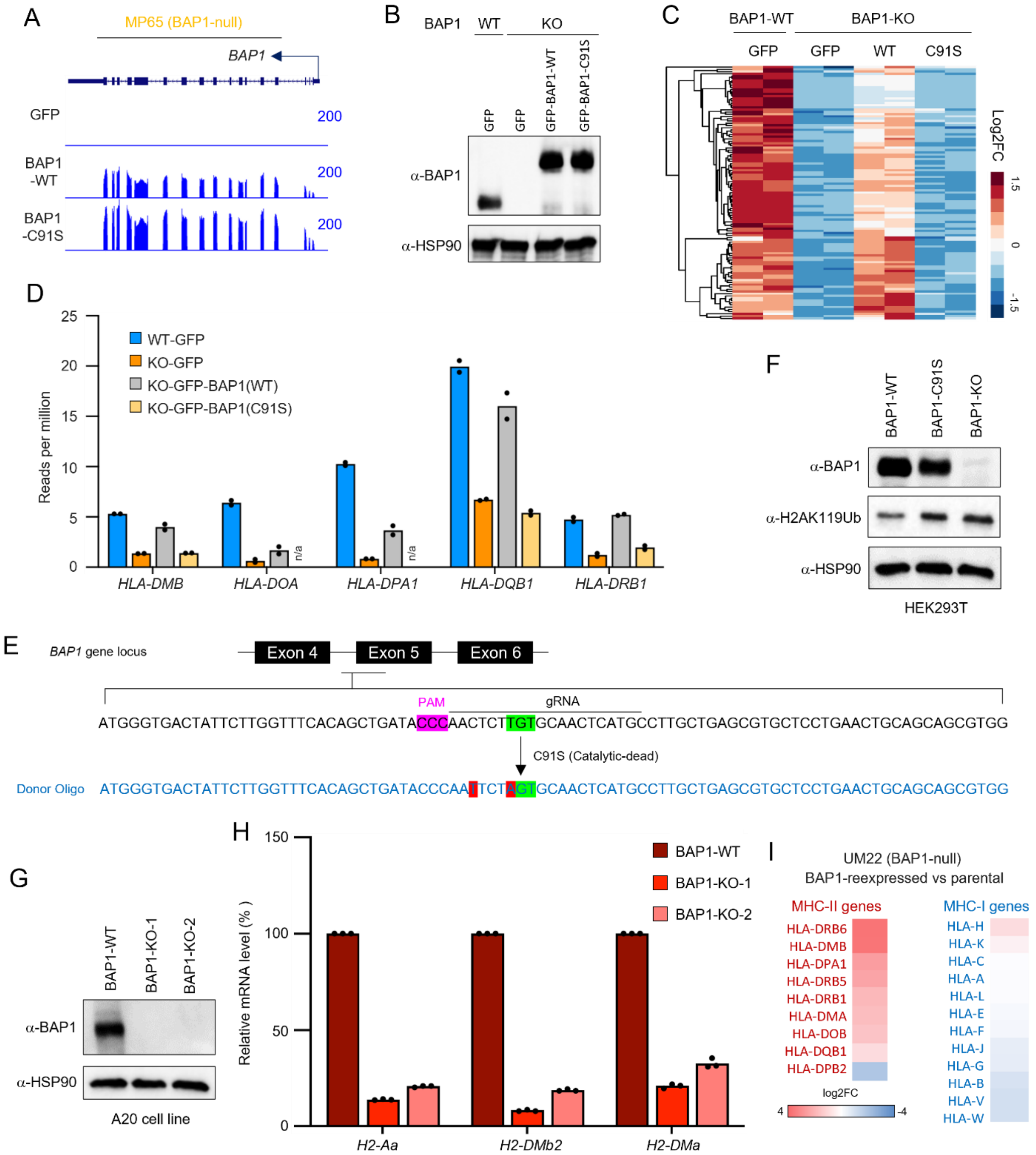

Supplemental Figure 1. BAP1 regulates MHC-II cluster genes in a catalytically dependent manner.

A) The track example shows the expression levels of BAP1 in MP65 cells transduced with either GFP, wild type BAP1, or catalytically dead BAP1. B) The HEK293T-BAP1-KO cells were

transduced with either GFP, wild type BAP1, or catalytically dead BAP1. The protein levels of BAP1 were determined by western blot. Representative blot from 2 biological repeats. C) The gene expression profiles in the above cell lines were determined by RNA-seq. D) The expression levels of MHC-II cluster genes in the above cell lines were shown by the bar plot. E) The schematic of human BAP1 gene locus and the CRISPR guide RNA designed to introduce the catalytically dead mutation C91S of BAP1. F) The protein levels of BAP1 and histone H2AK119Ub were determined by western blot in BAP1-WT, BAP1-C91S-knockin, and BAP1-KO HEK293T cells. Representative blot from 2 biological repeats. G) The protein levels of BAP1 were determined by western blot in wild type and BAP1-KO A20 cells. Representative blot from 2 biological repeats. H) The mRNA levels of MHC-II genes *H2-Aa*, *H2-DMb2*, and *H2-DMa* were determined by real time PCR in A20 BAP1-WT and -KO cells. n = 3 technical replicates. Data are represented as mean ± SD. I) The gene expression profiles of parental and BAP1 restored UM22 cell line was retrieved from public available database[41], the heatmap shows the log2 fold change of both MHC-I and MHC-II cluster genes between BAP1 null and BAP1-reexpressed cells.

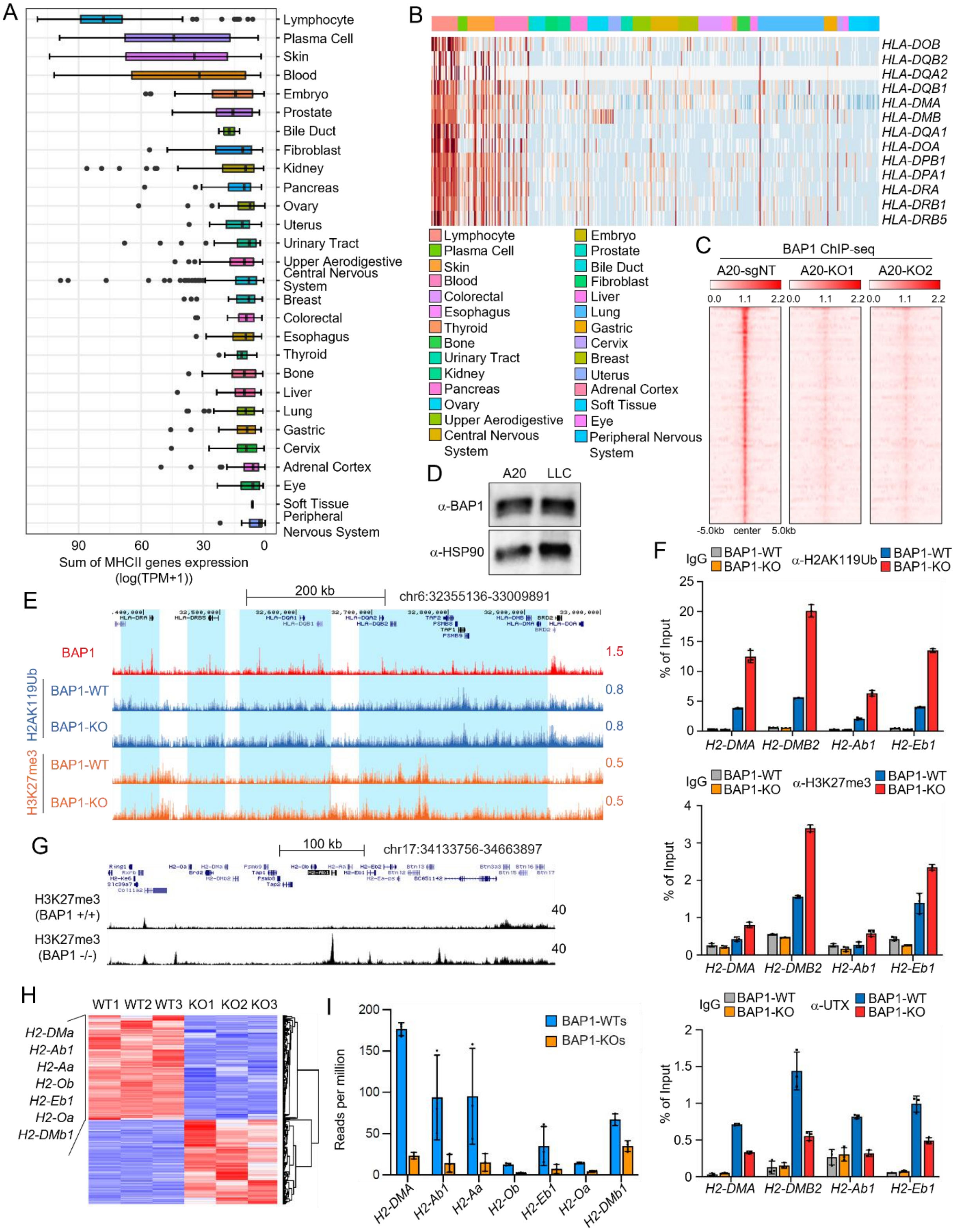

Supplemental Figure 2. BAP1 occupies MHC-II gene loci and governs chromatin states.

A-B) The gene expression profiles for 934 human cancer cell lines were retrieved from CCLE database. The sum and individual of MHC-II cluster genes in each cancer type were analyzed by the box plot (A) and heatmap (B). C) The specificity of ChIP-seq grade BAP1 antibody was validated by A20-BAP1-WT and two independent KO clones. D) The protein levels of BAP1 in A20 and LLC cells were determined by western blot. Representative blot from 2 biological repeats. E) The track example shows the BAP1 peaks and histone H2AK119Ub levels in BAP1-WT and -KO HEK293T cells at human MHC-II loci. F) The H2AK119Ub, H3K27me3, and UTX levels at MHC-II gene loci were determined by ChIP-QPCR in both A20 BAP1-WT and -KO cells. n = 3 technical replicates. Data are represented as mean ± SD. G, H) The RNA-seq data (GSE61577) and ChIP-seq data (GSE61360) were retrieved from this study on BAP1 deletion in mouse bone marrow cells. The track example in (G) shows the H3K27me3 levels in BAP1-WT and KO bone marrow cells. The heatmap (H) and bar plot (I) shows the expression levels of MHC-II genes between BAP1-WT and BAP1-KO bone marrow cells.

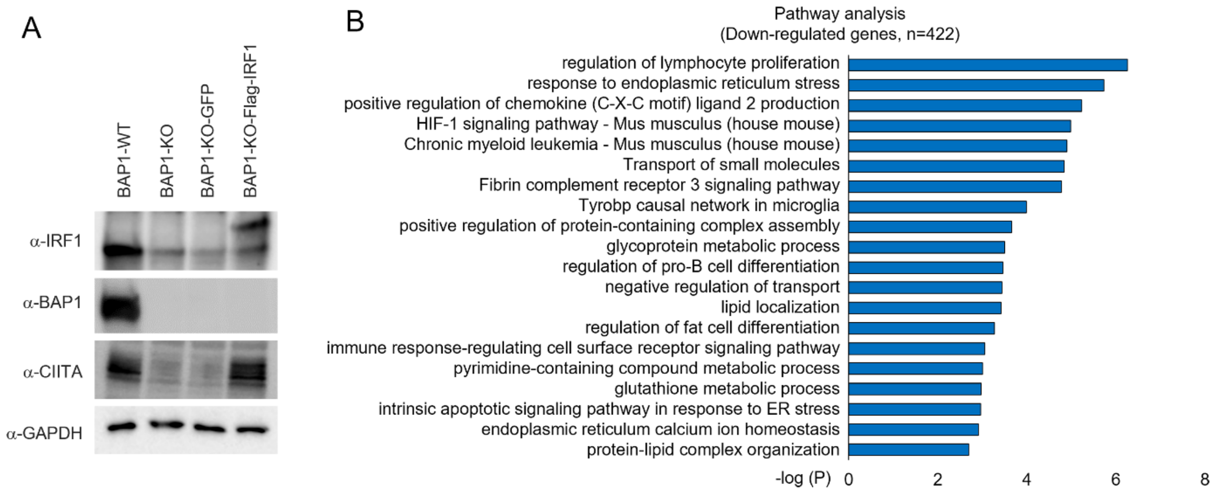

Supplemental Figure 3. Loss of BAP1 attenuates IRF1-dependent regulation of CIITA/MHC-II axis.

A) The A20 BAP1-KO cells were infected with lentivirus expressing GFP or GFP-tagged IRF1. The protein levels of BAP1, IRF1, and CIITA were determined by western blot. Representative blot from 2 biological repeats. B) The Pathway analysis with all the genes that are significantly down regulated in IRF1 restored BAP1-KO cells.

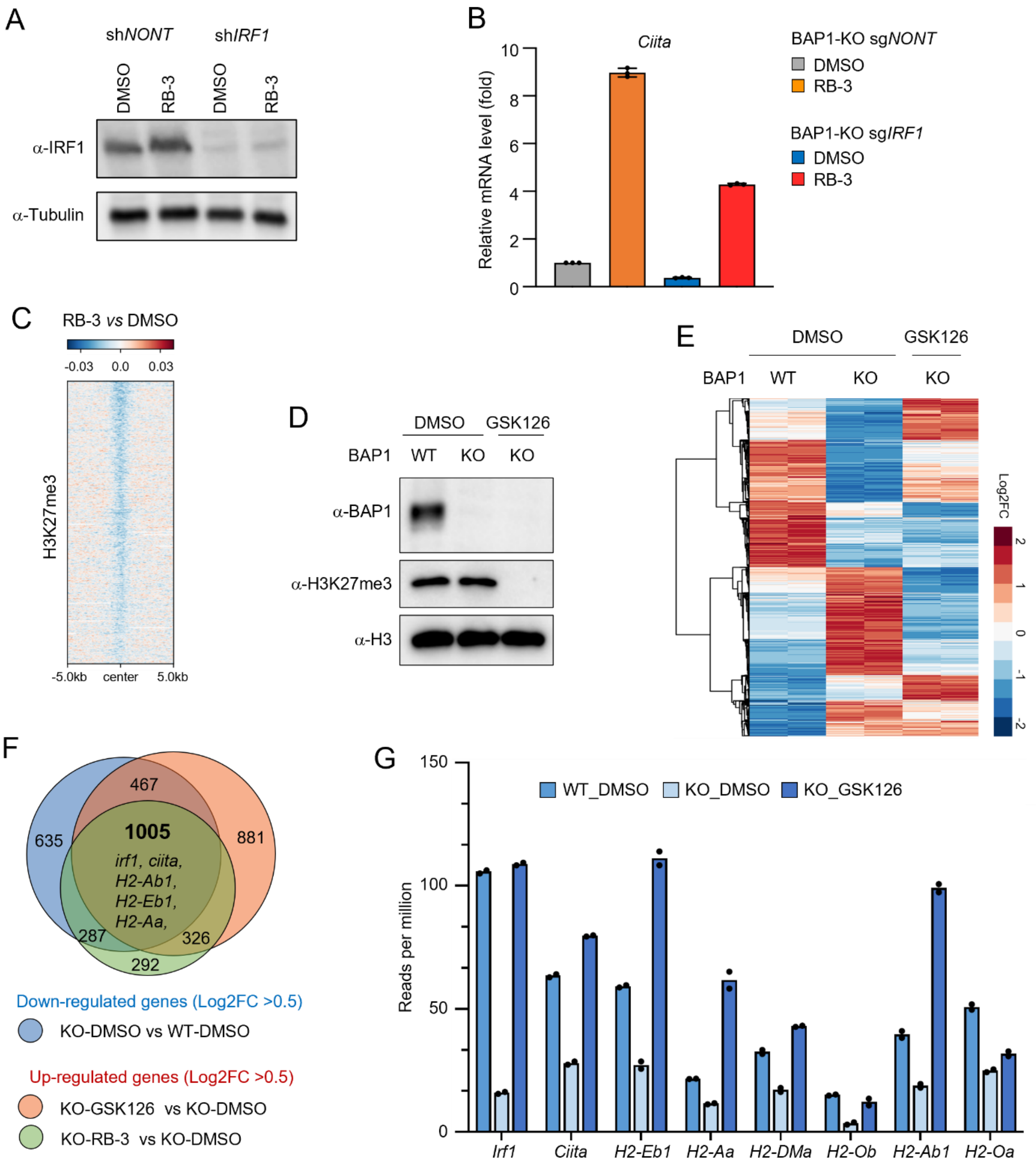

Supplemental Figure 4. Polycomb inhibition rescues IRF1-dependent MHC-II expression in BAP1 deficient B cell lymphoma.

The A20-BAP1-KO cells were infected with lentivirus expressing either non-targeting sgRNA or irf4-specific sgRNA, followed by RB-3 treatment. A) The protein levels of IRF1 were determined

by western blot. Representative blot from 2 biological repeats. B) The mRNA levels of Ciita were determined by real-time PCR in each condition. n = 3 technical replicates. Data are represented as mean ± SD. C) The A20-BAP1-KO cells were treated with PRC1 inhibitor RB-3 (5 μM) for 6 days. The log2 fold change heatmap shows the level of H3K27me3 in DMSO and RB-3 treated cells centered on BAP1 peaks. D) The A20-BAP1-WT cells were treated with DMSO for six days, and the BAP1-KO cells were treated with either DMSO or PRC2 inhibitor GSK126 (5 μM) for six days. The protein levels of H3K27me3 were determined by western blot. Representative blot from 2 biological repeats. E) The heatmap shows the gene expression profiles for each cell from (D). F) The Venn-diagram shows the overlapping genes between BAP1 target genes and GSK126 or RB-3 rescued genes in BAP1-KO cells. G) The bar plot shows the rescue of MHC-II cluster genes by GSK126 treatment in BAP1-KO cells.

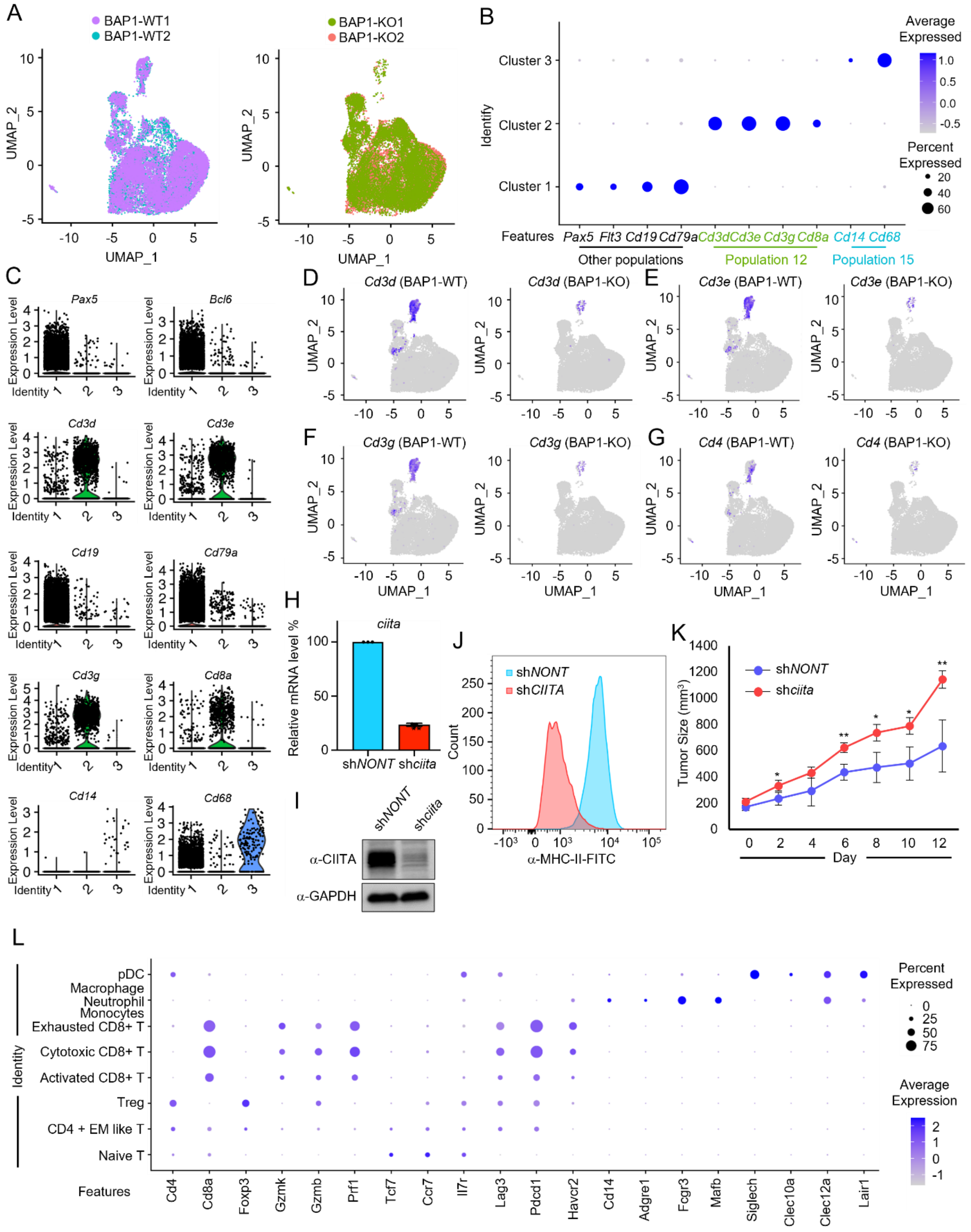

Supplemental Figure 5. Loss of BAP1 reduces immune cell infiltration and promotes B cell lymphoma growth *in vivo*.

A) The UMAP analysis shows the correlation between two biological replicates of BAP1-WT and -KO tumor tissues subjected to single cell RNA-seq. B) The gene expression signature shows the enrichment of B cell lymphoma markers (*Pax5*, *Flt3*, *Cd19*, and *Cd79a*), T cell markers (*Cd3d*, *Cd3e*, *Ce3g*, and *Cd8a*), and macrophage markers (*Cd14* and *Cd68*) in three major cell clusters- Cluster 1 (All populations except #12 and #15), Cluster 2 (#12, T cell population), and Cluster 3 (#15, macrophage population). C) The dot plot shows the most significant enriched genes in the three major cell clusters, B cell lymphoma (All the other populations expect #12 and #15), T cell (#12), and Macrophage (#15). The UMAP plot shows the *Cd3d* (D), *Cd3e* (E), *Cd3g* (F), and *Cd4* (G) cell populations between BAP1-WT and -KO tumor tissues. H-J) The wild type A20 cell line was infected by lentivirus expressing either nontargeting shRNA or CIITA specific shRNA. The mRNA levels of CIITA were confirmed by real time PCR (H). n = 3 technical replicates. Data are represented as mean ± SD. The protein levels of CIITA and MHC-II were confirmed either western blot (I) or FACS analysis (J). Representative blot from 2 biological repeats. K) 5×10^6 of A20 CIITA-WT and deficient cells were inoculated into the right flank of 6-week-old BALB/c mice, respectively. The tumor size was measured every other day two weeks after the inoculation. Data are represented as mean ± SEM. A two-tailed unpaired Student's t-test was used for statistical analysis. $^{**}P < 0.01$; $^{*}P < 0.05$. L) The gene expression signature shows the enrichment of genes in each immune cell population that was defined in Figure 5I.
