# Supplementary material for: An epigenetic pathway regulates MHC-II expression and function in B cell lymphoma models
Source: J Clin Invest. 2025 Jan 16;135(2):e179703. doi: 10.1172/JCI179703 (PMC11735100; doi:10.1172/JCI179703)

Full unedited gel for Figure 1

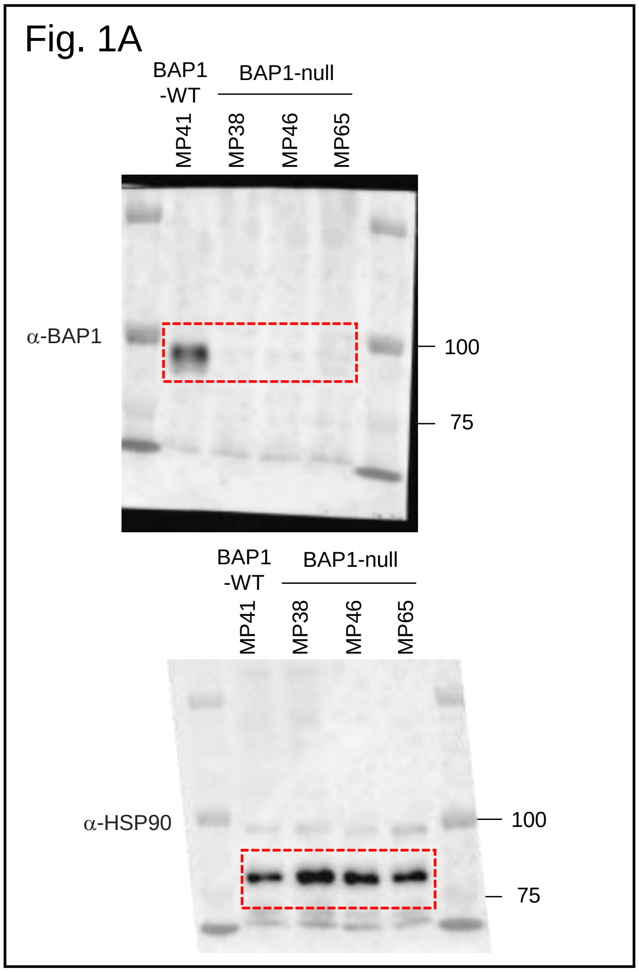

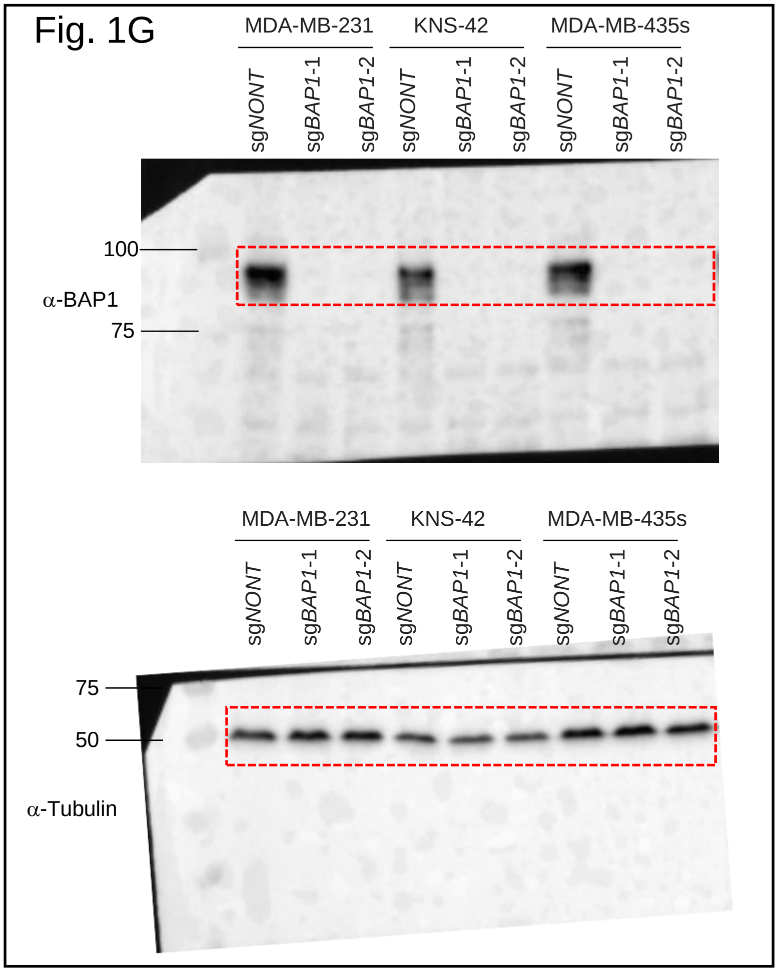

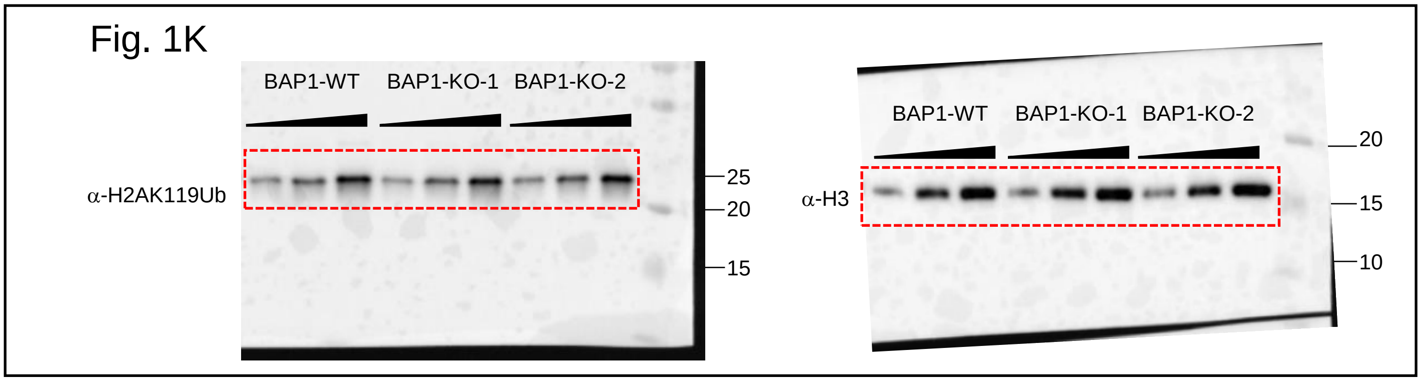

Full unedited gel for Figure 3

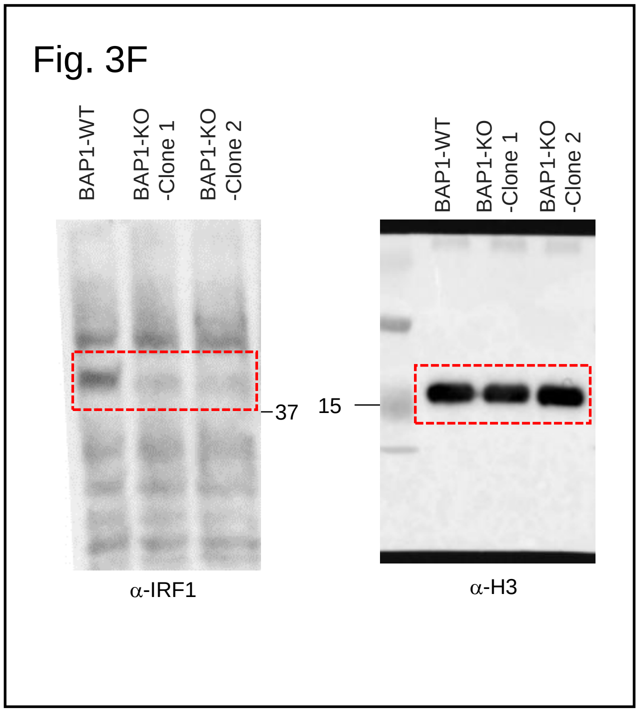

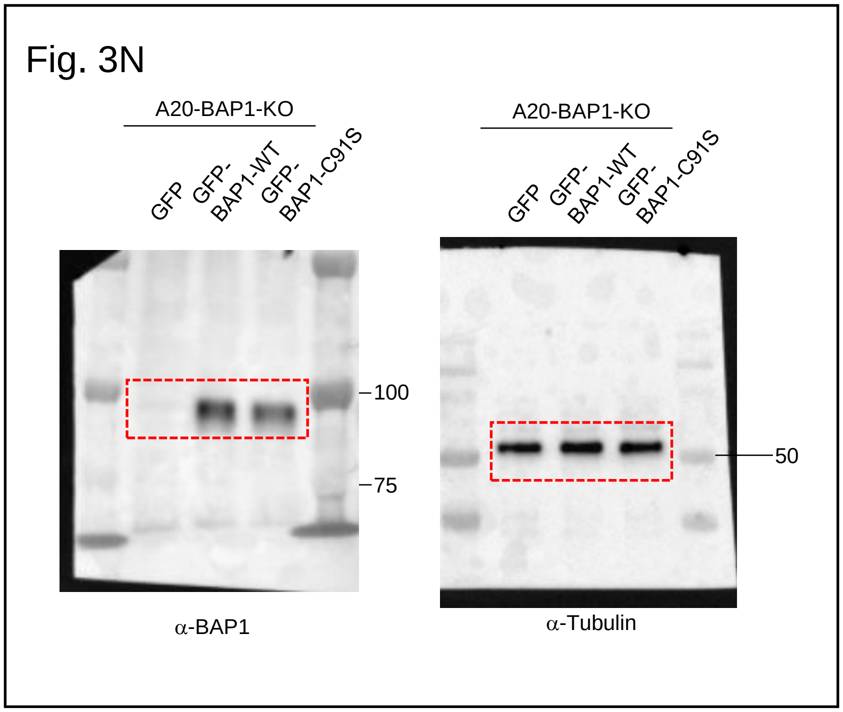

Full unedited gel for Figure 4

Fig. 4A

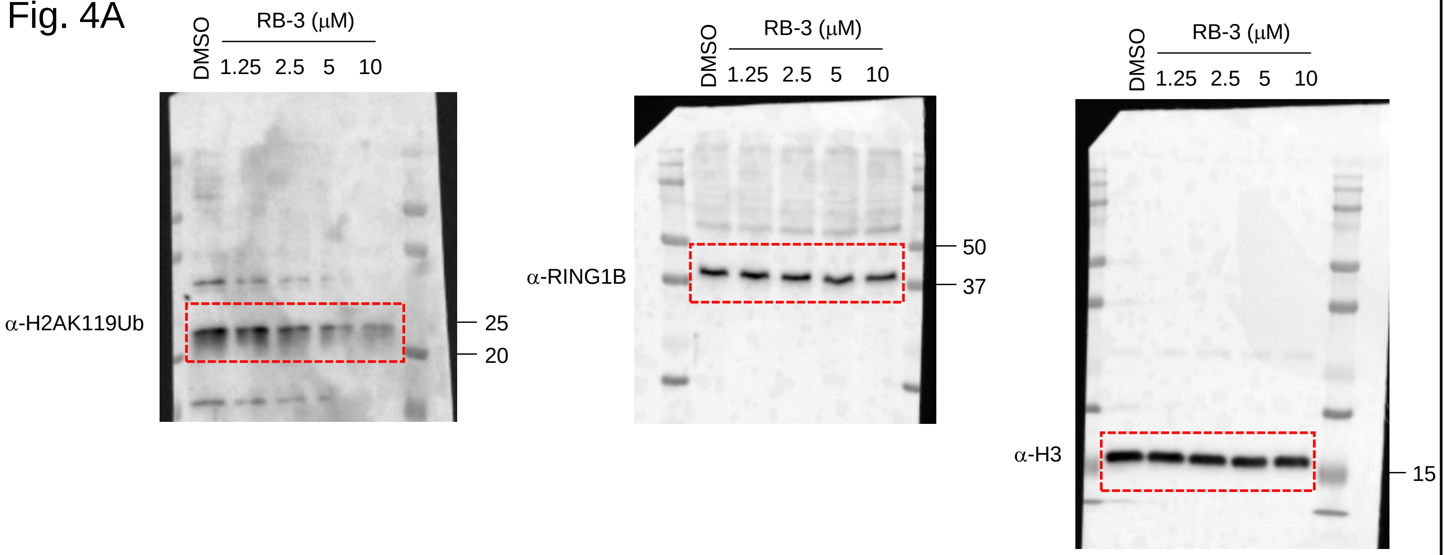

Fig. 4H

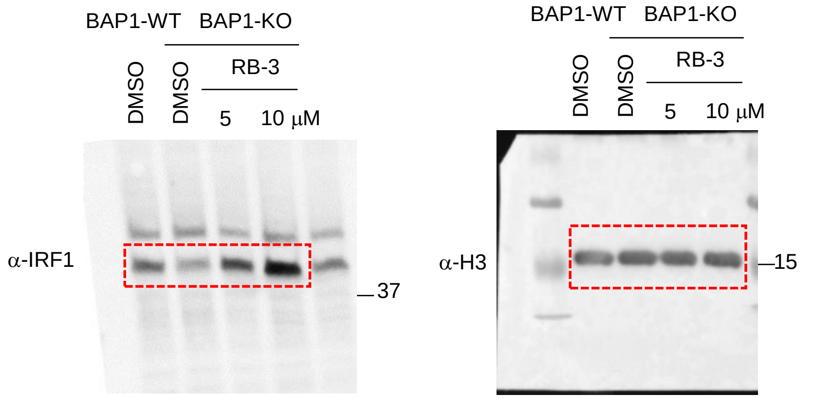

Fig. 4K

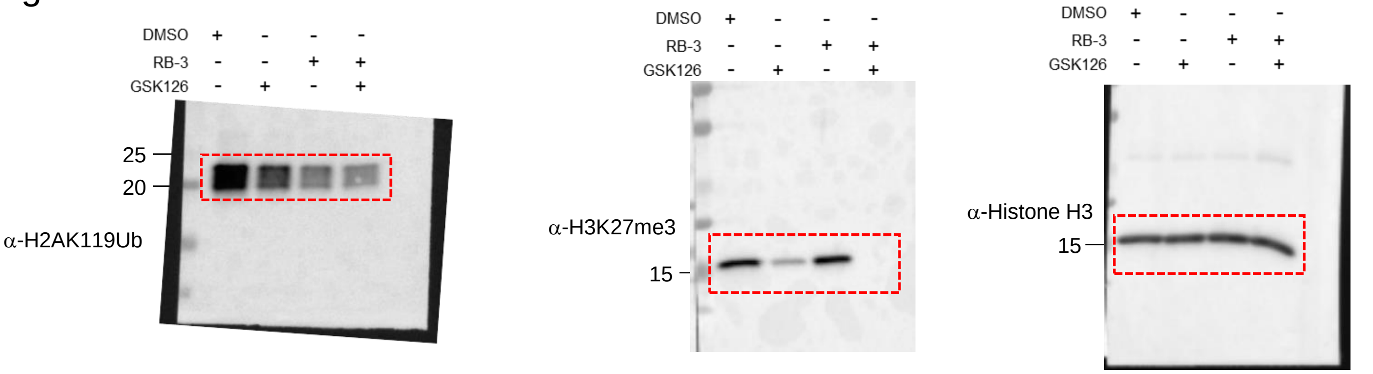

Full unedited gel for Supplemental Figure 1

Fig. S1B

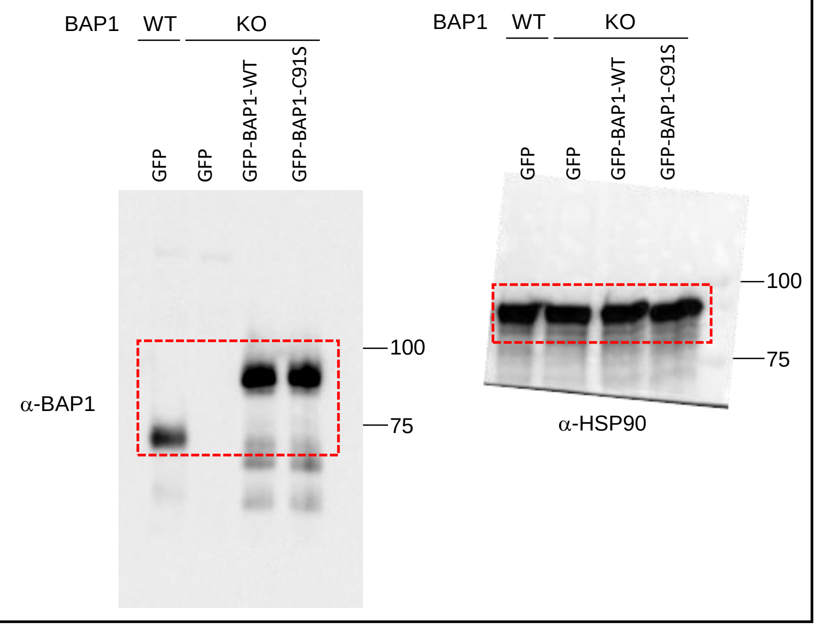

Fig. S2D

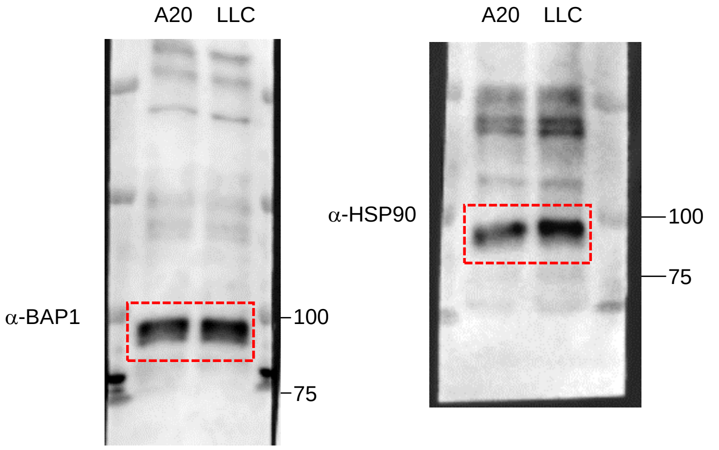

Fig. S1F

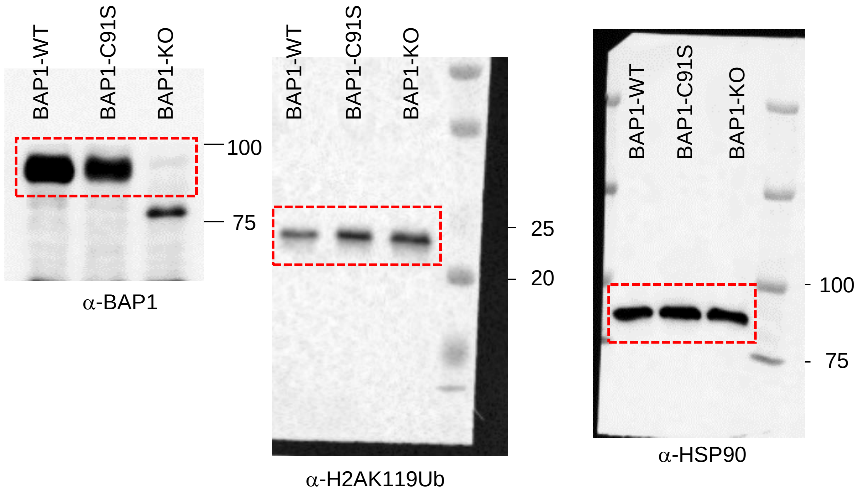

Fig. S1G

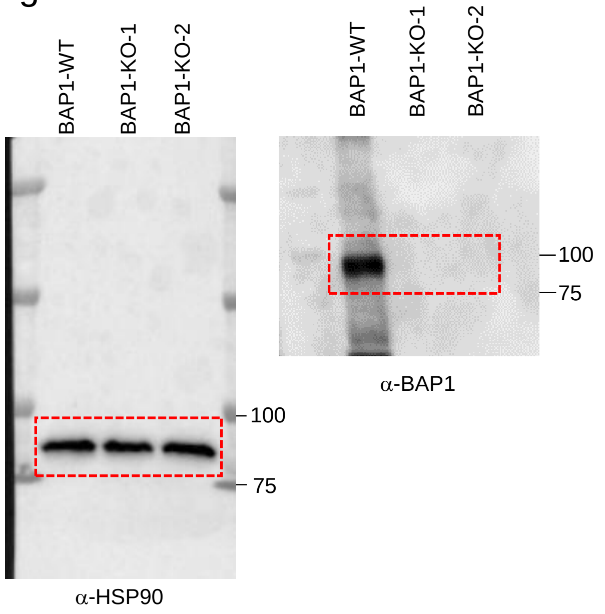

Full unedited gel for Supplemental Figure 3

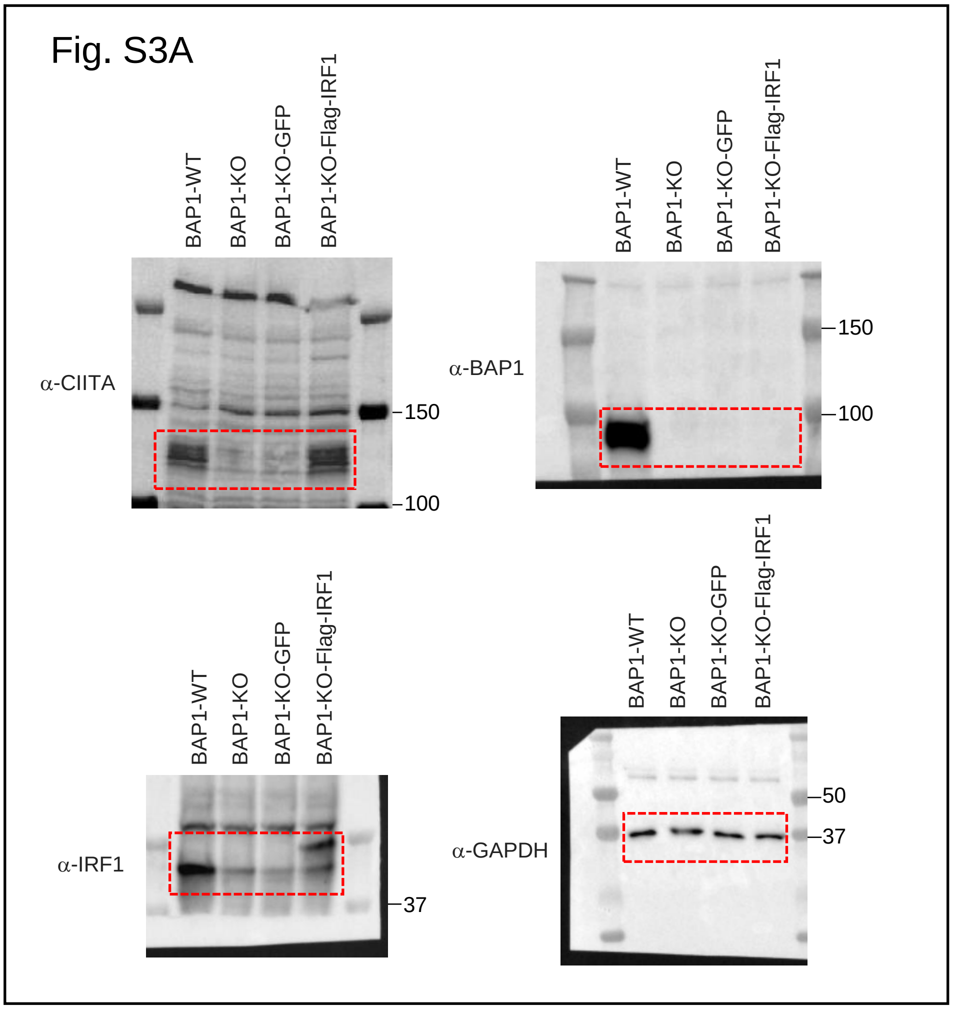

Full unedited gel for Supplemental Figure 4

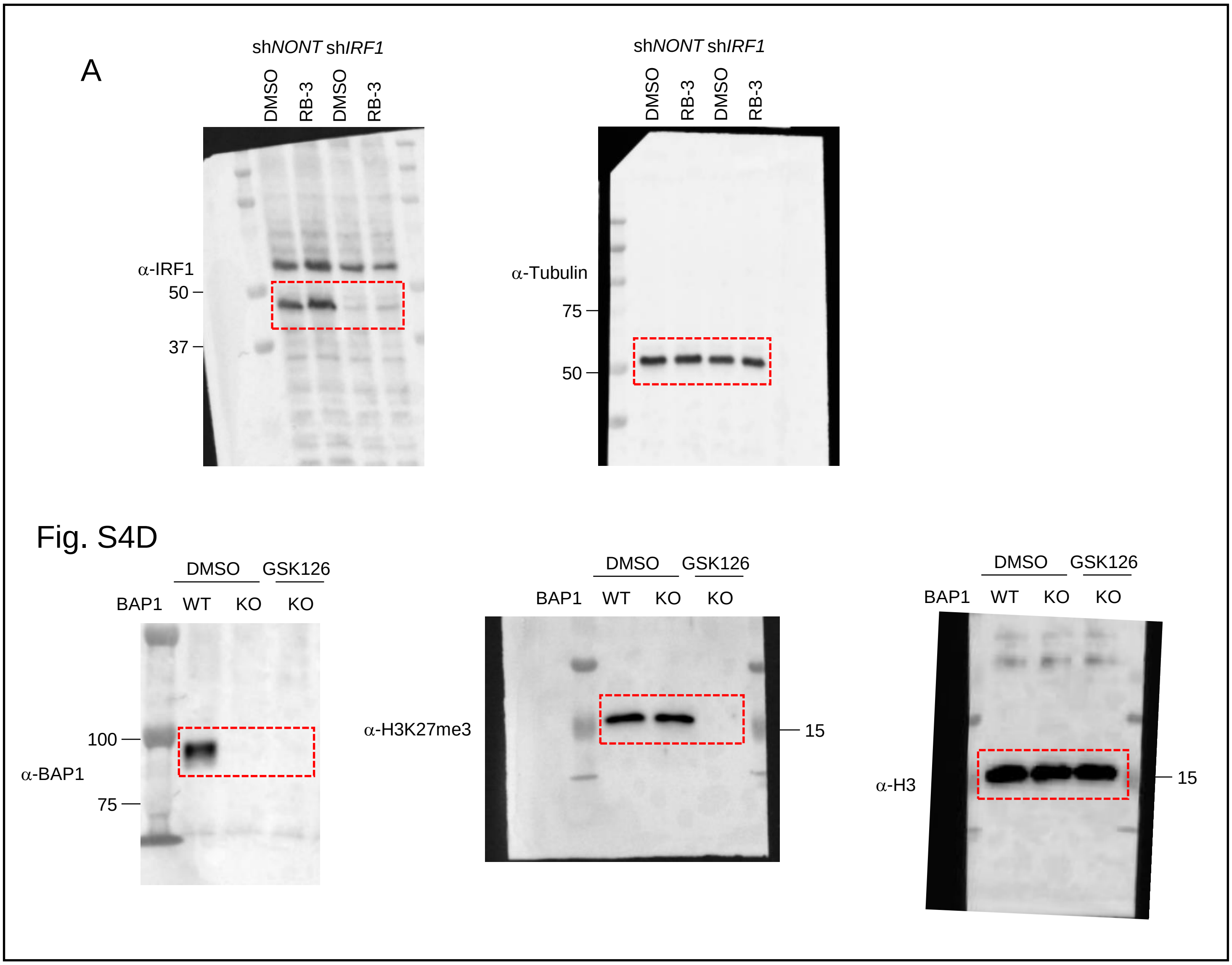

Full unedited gel for Supplemental Figure 5

Fig. S5I

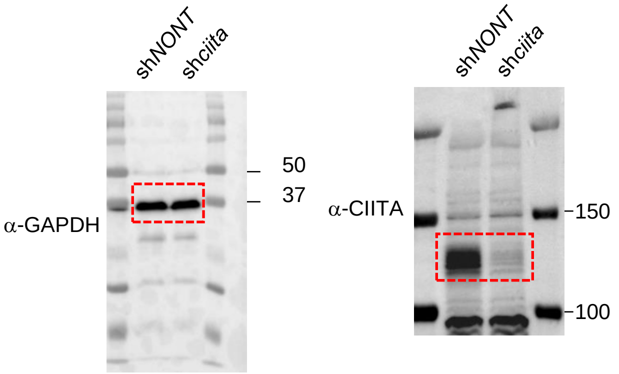

Supplement: Unedited blot and gel images [file jci-135-179703-s147.pdf]
